# Influence of Freeze-Dried Phenolic-Rich Plant Powders on the Bioactive Compounds Profile, Antioxidant Activity and Aroma of Different Types of Chocolates

**DOI:** 10.3390/molecules26227058

**Published:** 2021-11-22

**Authors:** Dorota Żyżelewicz, Joanna Oracz, Martyna Bilicka, Kamila Kulbat-Warycha, Elżbieta Klewicka

**Affiliations:** 1 Institute of Food Technology and Analysis, Faculty of Biotechnology and Food Sciences, Lodz University of Technology, 2/22 Stefanowskiego Street, 90-537 Łódź, Poland; joanna.oracz@p.lodz.pl (J.O.); martyna.bilicka@gmail.com (M.B.); kamila.kulbat-warycha@p.lodz.pl (K.K.-W.); 2 Institute of Fermentation Technology and Microbiology, Faculty of Biotechnology and Food Sciences, Lodz University of Technology, 171/173 Wólczańska Street, 90-530 Łódź, Poland; elzbieta.klewicka@p.lodz.pl

**Keywords:** antioxidants, chocolate, free radical scavenging activity, reducing power, functionalization of food, electronic nose analysis

## Abstract

In this study, the blueberries (BLUB), raspberries (RASB), blackberries (BLCB), pomegranates pomace (POME) and beetroots (BEET) freeze-dried powders were used as the sources of phenolic compounds to enrich different types of chocolates, substituting a part of the sweetener. It was found that 1% addition of fruit or vegetable powders to chocolates increased the content of total phenolic compounds (flavan-3-ols, phenolic acids and anthocyanins) of enriched dark and milk chocolates compared to the control ones dependent on the powder used. Among the enriched chocolates, the chocolates with the addition of BLUB powder were characterized by the highest total polyphenol content. The highest percentage increase (approximately 80%) in the total polyphenol content was observed in MCH chocolate enriched with BLUB powder. Chocolates incorporated with BLUB, RASB, BLCB and POME powders presented a richer phenolic compound profile than control counterparts. The highest DPPH radical-scavenging capacity was exhibited by the DCH98S chocolate enriched with BEET powder. However, the DCH98ESt chocolates enriched with POME and BEET powders demonstrated the highest FRAP values. An electronic nose analysis confirmed the existence of differences between the profiles of volatile compounds of various types of chocolates enriched with fruit or vegetable powders. Thus, the enrichment of dark and milk chocolates with BLUB, RASB, BLCB, POME and BEET powders seemed to be an interesting approach to enhance bioactivity and to enrich the sensory features of various chocolate types.

## 1. Introduction

In recent years, a rapid development of research on bioactive substances present in plant raw materials and their impact on the human body has been observed. Numerous studies revealed that there was a significant upward trend for the use of the plant-derived natural compounds as antioxidants and functional ingredients. Growing interest in the production and consumption of functional foods with specific pro-health characteristics results from the rationales indicating a close relationship between the consumption of food rich in natural antioxidants and the prevention of degenerative diseases. The main dietary sources of antioxidants are fruits and vegetables, as well as their derived products. The natural antioxidants occurring in plant materials are mainly phenolic compounds, carotenoids, and vitamins [1]. Many of these natural antioxidants, especially phenolic compounds, demonstrate pro-healthy properties. It was confirmed that the presence of these compounds in food plays an important role in the prevention of many civilization diseases, in particular cancer, cardiovascular diseases, as well as diabetes and rheumatoid arthritis [1,2,3]. However, the biological activity and bioavailability of these compounds highly depend on the molecular weight and chemical structure of these compounds, the matrix of food and their concentration in the consumed products [4].

Generally, cocoa bean and its derived products, such as chocolate and cocoa powder are a good source of natural antioxidants, especially phenolic compounds [5,6,7,8]. However, the production of chocolate may result in the loss of up to 80% of phenolic compounds originating from cocoa beans [9]. The largest and the most diverse group of phenolic compounds found in cocoa beans are flavonoids [5,10,11,12]. The predominant phenolic compounds in raw cocoa beans are proanthocyanidins (58%), monomeric flavan-3-ols (37%) and anthocyanins (4%) [10,11,13]. However, the level of these substances in cocoa beans can vary greatly and depends on a number of factors, mainly the variety, the geographical and environmental conditions during growth, the degree of ripeness, the harvest time, as well as the conditions and duration of storage after harvesting [14,15,16]. Many studies have shown that the processing of cocoa beans in order to obtain chocolate significantly influences the phenolic compounds content, and thus their activity and bioavailability [10,17,18,19,20,21]. All processing steps, such as fermentation, drying, alkalization and roasting, in addition to beneficial physicochemical, microbiological, and organoleptic changes, cause significant degradation of phenolic compounds [17,18]. During fermentation and drying of the cocoa beans, the phenolic compounds undergo biochemical transformations leading to a reduction in their content. Monomeric flavan-3-ols are enzymatically oxidized to semi-quinones and quinones. Anthocyanins, which are highly unstable and susceptible to various degradation reactions such as enzymatic or non-enzymatic browning, in the presence of the glycosidases are hydrolyzed to anthocyanidins and monosaccharides, mainly arabinose and galactose [13,15,19,22]. Phenolic compound content decreases with thermal processing. Heat exerted during the roasting causes degradation of these substances through non-enzymatic oxidation and polymerization reactions of proteins and protein hydrolysis products, as well as amino acids, polysaccharides and Maillard reaction products leading to the formation of insoluble macromolecular complexes [21,23]. As a result, the concentration of phenolic compounds in raw cocoa beans differs significantly from that in the roasted beans and chocolate [21,24,25,26].

In recent years, a healthy lifestyle has been promoted. It is mainly manifested by caring for health and taking up physical activity in order to ensure a longer life in a good physical and mental condition. One of the manifestations of caring for health is eating food that is not only of the highest nutritional value, but also has specific health-promoting properties. However, food processing can lead to a reduction in the concentration or even complete loss of many valuable bioactive ingredients positively influencing human health. Hence the efforts of food and nutrition technologists, dieticians and, consequently, many conscious food producers to functionalize (design food products) the aim of which is not only to provide all nutrients, but also to positively influence the body’s functions by providing compounds that reduce the risk of developing certain diseases, especially the so-called civilization diseases, or providing compounds that generally improve health and well-being. In the case of chocolate production, the content of bioactive ingredients, such as phenolic compounds, is also reduced. Already at the stage of fermentation of cocoa beans in plantations, their content is reduced by over 50%. Especially anthocyanins and (−)-epicatechin are highly degraded. Drying the beans after fermentation causes a further reduction in the concentration of these compounds. Some authors [5] state that up to 90% of (−)-epicatechin is lost as a result of fermentation and drying of cocoa beans. Further processes leading to obtaining chocolate, especially roasting cocoa beans, only increase the loss of polyphenols. That is why scientists, food technologists and dieticians see the need to enrich food, including chocolate, with an additional portion of bioactive compounds, including antioxidants, and the functional food market is constantly growing and not only small enterprises with niche production but also industrial giants are investing in it [5,16]. The current scientific literature on the subject describes few proposals in this area, starting from the introduction of an additional portion of polyphenols in pure form, in the form of cocoa liquor from raw beans, cocoa liquor or cocoa powder with increased polyphenol content [27,28,29] to the addition of spices [30], leaves [31,32] or pomaces [33]. These studies have shown that in this way the polyphenol content and antioxidant activity of chocolates can be increased and their sensory profile can be influenced. There are commercially available chocolates with low or unprocessed fruits, e.g., almonds, nuts, raisins, but their shelf life is shorter than chocolate without such additives, due to, among other things, a higher water content in semi-finished fruit products (raisins) or a high content of unsaturated fatty acids (nuts, almonds) [34]. The introduction of freeze-dried fruit or vegetable powder into chocolate recipes eliminates this inconvenience. To the authors’ knowledge, no studies have been carried out in terms of the aromatic profile, polyphenol content and antioxidant activity of different kinds of chocolates enriched with freeze-dried fruit or vegetable powders. For this purpose, we used several types of berries (blueberries, raspberries, blackberries), pomegranate pomace and beetroots and we assumed the aim of the presented research to investigate the effect of phenolic-rich plant powders fortification on the bioactive compounds profile, antioxidant activity and aroma (measured by an objective method using an electronic nose) of different types of chocolates. Berries and pomegranates contain significant amounts of flavonoids, mainly anthocyanins, and possess many beneficial properties for human health [35]. Anthocyanins are considered as strong antioxidants and free radical scavengers that have an antioxidant potential twice as high as that of other antioxidants, such as (+)-catechin and α-tocopherol [36]. Therefore, these fruits could be used as a source of phenolic substances with pronounced antioxidant activity. Powdered dried fruits (including berries) and vegetables are new and valuable ingredients for the chocolate industry. European Union regulations allow the introduction of up to 40% of additional foodstuffs to chocolate recipes [37]. In this context, the enrichment of chocolates with freeze-dried fruit (berries, pomegranate) or vegetable (beetroots) powder, which is a rich source of natural antioxidants, including anthocyanins, can greatly improve functional and health-promoting properties of these products. However, the addition of plant powders rich in anthocyanins or other phenolic compounds may have an effect on the organoleptic properties, including aroma, and in consequence consumer acceptance of these novel chocolate products, depending on the source of the bioactive compounds and the type of product in which they are contained.

## 2. Results and Discussion

### 2.1. Water Content and Activity and Color of Chocolates

Table 1 shows the water content and activity, as well as the CIE L*a*b* and organoleptic characteristics of the tested chocolates.

Water content is one of the quality parameters of chocolate that affects the properties of this product. The increase in the water content causes the deterioration of the rheological properties of the chocolate. This influences, among other things, an increase in the power consumption of devices during production, problems with pumping of the chocolate mass, coating of cores, forming the product into bars or figures and worse sensory perceptions during its consumption (chocolate sticks to the teeth). The water content of the chocolates should be as low as possible. Bolenz and Glöde (2021) indicate that it should be lower than 0.6% [33]. This is difficult to achieve, especially in the case of white or milk chocolates due to the higher water content in milk powder than in other raw materials used in the production of chocolate. Problems with achieving such a low value of this parameter may also occur in the production of sugar-free chocolates. Sucrose substitutes are often characterized by a higher water content than sugar (sucrose), hence the generally higher moisture content of this type of product. Thus, in practice, it is often aimed at less than 2%. The water content in chocolates also depends on the production technology used, and the type and design of the machines used.

Our results are consistent with the results of other researchers. An example is the study by Godočiková et al. (2019) who studied the antioxidant properties and volatile profile of different kinds and types of chocolates produced in Slovakia with various fruit and nut additions [34]. They found the water content in dark chocolates at 1.1%, in milk chocolates at 1.8% and in white chocolates at 1.6%. Chocolates enriched with plant additives (sea buckthorn, almonds, mulberry, currant, cherry) were characterized by a much higher value of this parameter (8.7–16.6%). In our studies the water content in different types of chocolates varied from 0.93 to 2.19%, with the differences being statistically significant (*p* < 0.05).

Our results indicated that the type of chocolate and functional enrichment affected the water activity of chocolates significantly (*p* < 0.05). The water activity of tested chocolates ranges from 0.378 to 0.490. The present results are within the range reported by other authors [28,34]. Looking at the effect of freeze-dried fruit or vegetable powder on the water activity of chocolates, it was demonstrated that a partial replacement of sweetener in different types of chocolates by 1% freeze-dried phenolic-rich plant powder did not cause an increase of the water activity above the optimal level. Good quality and microbiologically stable chocolates are characterized by low moisture content and water activity of 0.25–0.50 [38]. Interestingly, it can be observed that some chocolate samples enriched with freeze-dried fruit or vegetable powder had lower moisture content and water activity than control ones. The differences in water content and activity could be explained with the changes in the chemical composition of the control and supplemented chocolates.

The color, next to the characteristic chocolate aroma and taste, is one of the basic attributes influencing the quality of chocolate products. These parameters were dependent on the type of chocolate. In our case, it was influenced by such factors as the content of cocoa liquor and related to this sweetener content, presence of freeze-dried plant powder, as well as the presence of powdered milk in milk chocolates. As can be seen in Table 1, the type of chocolate affected the CIE L*a*b* color parameters of the chocolates significantly (*p* < 0.05). The brightness (L*) of the surface of tested chocolates ranged from 27.22 to 34.01, while the redness (a*) and yellowness (b*) were 3.03–7.99 and 0.93–2.19, respectively. The results suggested that the control chocolates and the supplemented chocolates differed (*p* < 0.05) in terms of values of parameters L*, a* and b*. However, the values of parameter b* remained more similar. Generally, the addition of freeze-dried phenolic-rich plant powder to DCH, DCH98S, DCH98ESt or MCH significantly (*p* < 0.05) brightened the color on the surface of enriched chocolates compared to the control ones, which could be attributed to some changes in the crystal structure of cocoa butter (polymorphism) [32]. On the other hand, there were only slight differences for the a* and b* parameters between the control chocolates and the enriched chocolates of the same type. These results suggested that the addition of 1% freeze-dried phenolic-rich plant powder has no negative effect on the color of the enriched chocolates. Similar results were presented by Muhammad et al. (2018) for milk chocolates supplemented with cinnamon nanoparticles [30].

The organoleptic evaluation of the tested chocolates was in the range of 3.8–4.9 points (Table 1). The DCH chocolates were rated the best, followed by the MCH ones, and the worst were the DCH98Est. The organoleptic quality of the DCH and the MCH chocolates, control and with the addition of BLUB, RASB, BLCB and POME powders was rated as extremely desired. They obtained scores from 4.6 to 4.9 points, while the scores within one group of chocolates, i.e., the DCH or the MCH group, did not differ significantly from each other (*p* ≥ 0.05). For example, in the case of the DCH chocolates, their rating was ranging from 4.7 to 4.9 points. Chocolates with BEET powder were rated the worst, though still in the desired organoleptic quality category. This was due to the earthy taste of the chocolates, which was especially noticeable in the case of milk chocolate (3.8 points). Among dark chocolates, chocolates with 98% cocoa were rated worse than the DCH chocolate. Panelists described them as tart and slightly more acidic than the DCH chocolates.

Two-way ANOVA revealed a significant effect of chocolate type (*p* < 0.001), phenolic-rich plant powder (*p* < 0.05) and their interaction (*p* < 0.01) for the water content and activity, the CIE L*a*b* color parameters and organoleptic characteristic of the tested chocolates (Table S1, Supplementary Materials).

### 2.2. Profile and Concentrations of Bioactive Compounds

The results show that there was considerable diversity between the profile and the levels of bioactive compounds in the control and the enriched chocolates. It is worthwhile noting that raw cocoa beans are known to be a good source of phenolic compounds, mainly flavonoids, but most of them are lost during the production of chocolate and thus even dark chocolate, which contains more cocoa parts than milk chocolate, has a significantly lower level of phenolic compounds. As a result of fermentation and drying, the concentration of phenolic compounds, especially flavonoids, decreases by more than 80% [26]. In the case of unstable anthocyanins, the losses may reach even up to 90% [10]. Heat treatment may also cause the transformation of the remaining anthocyanins into colorless chalcones, which spontaneously degrade to suitable phenolic acids or polymerize and condense with other phenolic compounds to form brown polymeric pigments. Therefore, chocolates obtained in the conventional processing of cocoa beans contain significantly less flavan-3-ols and do not contain anthocyanins. To minimize these losses, various plant extracts (e.g., raspberry leaf and green tea extracts) or dried fruits (e.g., cherry, black mulberry, currant, sea buckthorn) rich in phenolic compounds have been added to the white, milk or dark chocolates for enhancing their functional properties [27,30,31]. The application of freeze-dried fruit (blueberries, raspberries, blackberry, pomegranates pomace) and beetroot powder was exploited to enrich different kinds of chocolates, such as dark (DCH, DCH98S and DCH98ESt) and milk (MCH) chocolates, enhancing their health-promoting properties.

The addition of tested freeze-dried fruit or vegetable powders to different types of chocolates led to a significant (*p* < 0.05) enrichment of these products with phenolic compounds, mainly anthocyanins and phenolic acids (Table 2). It was found that a 1% addition of these powders to chocolates increased the content of total phenolic compounds (flavan-3-ols, phenolic acids and anthocyanins) of enriched dark and milk chocolates compared to the control ones dependent on the powder used. The highest percentage increase (approximately 80%) in the total polyphenol content was observed in the MCH chocolate enriched with BLUB powder (Table 3). The greatest increase in the total content of polyphenols in all obtained chocolates was caused by the addition of BLUB powder followed by the addition of BEET powder. The BLUB powder, in all obtained chocolates, enriched the polyphenol composition by approximately 32.8 mg/100 g DM of anthocyanins, which were absent in the control chocolates. The greatest amount of flavan-3-ols and phenolic acids was introduced into the chocolates also via BLUB powder. Therefore, the profile and concentrations of phenolic compounds in chocolates containing tested freeze-dried plant powders are influenced by the phytochemical composition of cocoa liquor and plant powder used as a functional additive. The contents of individual phenolic compounds detected in the control chocolates and in the chocolates made with 1% addition of the fruit or vegetable powder in the product recipe are presented in Table 3 (powders were added instead of part of sweetener).

The addition of plant powders made the chocolates differ in the total phenolics content and the qualitative and quantitative composition of polyphenols (Table 3). The feature that distinguished them is primarily the content and concentration of anthocyanins. The control and chocolates with BEET powder did not contain anthocyanins. Chocolates with the addition of BLUB powder included in their composition four anthocyanins: cyanidin-3,5-*O*-diglucoside (Cy-3,5-diGlu), cyanidin-3-*O*-xyloside (Cy-3-Xyl), delphinidin-3,5-*O*-diglucoside (Del-3,5-diGlu) and pelargonidin-3,5-*O*-diglucoside (Pel-3,5-diGlu), whose total concentration was approximately 33 mg/100 g DM. Cy-3,5-di Glu and Pel-3,5-diGlu were present in the highest amounts, each at concentrations of above 10 mg/100 g DM. Chocolates with the addition of RASB powder included only one anthocyanin—cyanidin-3-*O*-glucoside (Cy-3-Glu) in a concentration of approximately 3.7 mg/100 g DM. There were four anthocyanins in the chocolates with BLCB powder, i.e., Cy-3-Glu, cyanidin-3-*O*-rutinoside (Cy-3-Rut), Cy-3-Xyl and cyanidin-3-(6″-malonyl)-glucoside (Cy-3-(6″-Mal-Glu)), the total concentration of which was approximately 5 mg/100 g DM, with Cy-3-Glu being the highest amount of 4.4 mg/100 g DM. On the other hand, in chocolates with POME powder there were three anthocyanins, i.e., Cy-3,5-diGlu, Del-3,5-diGlu and delphinidin-3-*O*-glucoside (Del-3-Glu), with a total concentration of 0.14 mg/100 g DM, with Cy-3,5-diGlu being the highest amount of 0.1 mg/100 g DM.

Seven phenolic compounds were identified in all types of control chocolates and chocolates enriched with BEET powder, including four flavan-3-ols (catechin—Cat, epicatechin—Ecat, procyanidin B2—PC B2 and procyanidin C1—PC C1) and three phenolic acids (gallic acid—GA, protocatechuic acid—PA and *p*-hydroxybenzoic acid—*p*-HBA). The presence of these compounds in chocolates has already been described by other authors [29,39,40]. Chocolates enriched with RASB powder showed eight phenolic compounds, seven of which were present in the control chocolates and in chocolates made with vegetable powder in addition to Cy-3-Glu. The dark and milk chocolates containing BLCB powder showed the presence of eleven phenolic compounds and eight of these phenolics were identified in raspberry-enriched chocolates in addition to Cy-3-Rut, Cy-3-Xyl and Cy-3-(6″-Mal-Glu). The samples of DCH, DCH98S, DCH98Est and MCH made with BLUB powder also showed eleven phenolic compounds, eight of which were present in chocolates with BLCB powder in addition to Cy-3,5-diGlu, Del-3,5-diGlu and Pel-3,5-diGlu. The different types of chocolates containing POME powder showed the presence of ten phenolic compounds and nine of these phenolics were identified in BLUB-enriched chocolates in addition to Del-3-Glu. The predominant phenolic compound in all tested chocolates was Ecat. The second most abundant compound was PC B2, followed by Cat, PC C1, GA and *p*-HBA. Moreover, Cy-3,5-diGlu, Pel-3,5-diGlu, Cy-3-Glu and Cy-3-Xyl were found in significant quantities but only in chocolates made with berry powders. Depending on the evaluated chocolate types and functional enrichment, the concentrations of individual phenolic compounds varied significantly (*p* < 0.05) within tested chocolates.

As can be seen in Table 3, the sum of phenolic compounds differed significantly (*p* < 0.05) between control and enriched chocolates of different types, with the highest total phenolics found for DCH98S chocolate enriched with BLUB powder and the lowest for control MCH (the results ranged from 89.24 to 358.92 mg/100 g DM).

The major phenolic compounds in both control and chocolates enriched with fruit or vegetable powders were flavan-3-ols, which represented approximately 72–92% of total phenolics levels. The total amount of the investigated flavan-3-ols within the different chocolate types ranged from 79.66 mg/100 g DM in control milk chocolate to 294.82 mg/100 g DM in DCH98S chocolate enriched with BEET powder. The content of these compounds was similar to the amount reported by other authors [29,39,40] in different kinds of chocolates. Considerable amounts of phenolic acids were also found in tested chocolates. The concentrations of these phenolics represented approximately 8–11% of the average amounts of total phenolics. Among investigated samples, the highest phenolic acids levels were found in DCH98S chocolate enriched with either BLUB or POME powders. In turn, significant quantities of anthocyanins were found in chocolates enriched with fruit powders but mainly in those made with berry powders. Depending on the evaluated chocolate types and functional enrichment, the concentrations of anthocyanins varied significantly (*p* < 0.05) within chocolates enriched with fruit powders and ranged from 0.14 to 32.99 mg/100 g DM. In this study, no anthocyanins were found in the control chocolates. Moreover, chocolates with the addition of POME powder were characterized by very small amounts of these compounds.

The results indicated that the addition of all fruit and vegetable powders to different types of chocolates led to substantial changes in the levels of phenolic compounds of enriched chocolates (Table 3). A two-way ANOVA revealed that the content of all phenolic compounds, apart from anthocyanins, varied significantly with the type of chocolate (*p* < 0.001), phenolic-rich plant powder (*p* < 0.001) and their interaction (*p* < 0.001) (Table S2, Supplementary Materials). In addition, the total anthocyanins, Cy-3-Glu, Cy-3-Rut, Cy-3,5-diGlu, Cy-3-Xyl, Cy-3-(6″-Mal-Glu), Del-3,5-diGlu and Del-3-Glu concentrations were significantly affected by phenolic-rich plant powder, but there was not a statistically significant interaction between the effects of the type of chocolate and the phenolic-rich plant powder. The addition of all tested fruit and vegetable powders increased considerably the total content of phenolic compounds of all chocolate types compared to the control ones. Irrespective of the chocolate type, there was a significant increase in the level of flavan-3-ols and phenolic acids of the enriched chocolates. It was also observed that the addition of berry powders also caused a significant (*p* < 0.05) increase in the total anthocyanins content in both dark and milk chocolates with respect to control chocolates, while the amount of these pigments in chocolates made with POME powder only slightly increased compared to control ones. Overall, the greatest increment in the total contents of three classes of phenolic compounds of all types of chocolates was caused by the supplementation of BLUB powder. The increase in phenolic contents in fruit- or vegetable-supplemented chocolates reflects the addition of specific functional enrichment. Godočiková et al. (2017) reported also that specific types of dried fruits, for example black mulberry, rich in anthocyanins, was also suitable to enhance the concentration of bioactive substances of chocolate even with a lower cocoa solids content [41]. Recently, Martini, Conte and Tagliazucchi (2018) demonstrated that the enrichment of dark chocolates with Sakura green tea leaves or turmeric powder is an effective technique to improve the health-enhancement of the final product [42].

Our data supported the possible application of berries and POME powders to the formulation of both dark and milk chocolates with increased phenolic compounds, mainly anthocyanins, which are never found in a given type of cocoa bean or are lost during their processing.

### 2.3. Antioxidant Activity

**Figure 1:**
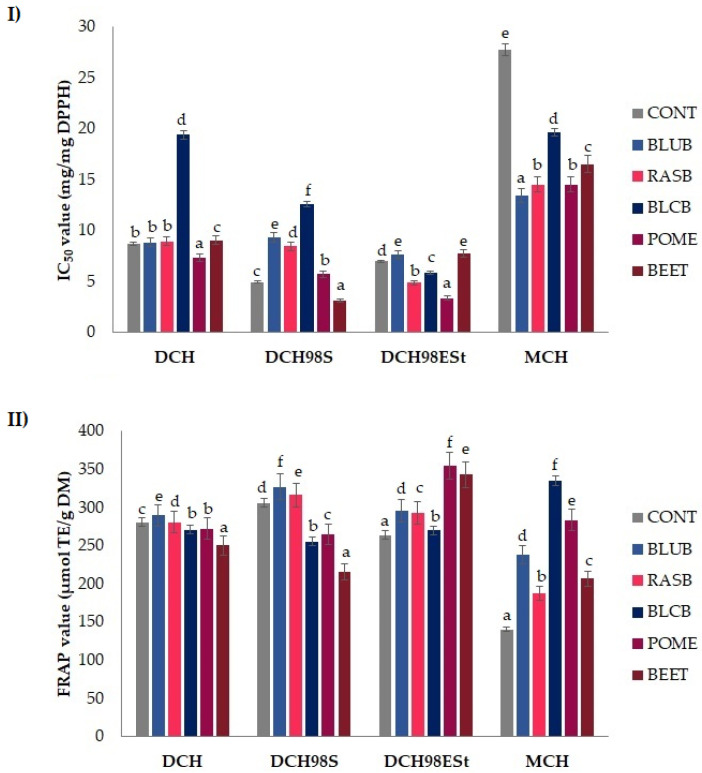
(**I**) DPPH radical scavenging activity of different types of chocolates enriched with various freeze-dried phenolic-rich plant powders, expressed as IC50 values. (**II**) Ferric reducing antioxidant power (FRAP value) of different types of chocolates enriched with various freeze-dried phenolic-rich plant powders. Data are expressed as the mean of triplicate ± SD. Bars with the same lowercase letter (a–f) within each type of chocolate do not differ significantly according to Tukey’s HSD test at *p* < 0.05. Blueberries (BLUB), raspberries (RASB), blackberries (BLCB), pomegranates pomace (POME), beetroots (BEET), control (CONT).

In order to evaluate the freeze-dried phenolic-rich plant powders’ contribution to the antioxidant properties of the different types of chocolates, the DPPH radical scavenging activity and the ferric-reducing ability in control and enriched chocolates were determined and the results have been presented in Figure 1I and II, respectively.

The DPPH• scavenging capacity was expressed as IC_50_ values, the concentration at which 50% inhibition of free radical scavenging activity is observed. The range of IC_50_ values of the analyzed chocolates was 3.05–28.12 mg/mg DPPH (Figure 1I). Two-way ANOVA revealed the significant effect of chocolate type (*p* < 0.001), freeze-dried phenolic-rich plant powders (*p* < 0.001) and their interaction for the DPPH radical scavenging activity (Table S2).

Overall, dark chocolates revealed better antioxidant properties than milk chocolates, which agrees with the higher content of phenolic compounds found in the dark chocolates. The antioxidant activity of chocolates is usually attributed to the presence of monomeric flavan-3-ols, polymeric procyanidins and Maillard reaction products (e.g., melanoidins) that are well known to possess effective scavenging activity of free radicals [43].

The results indicated that the enrichment of chocolates with various fruit or vegetable powders can improve the antioxidant activity of chocolates, depending on the chocolate type and plant powder used as a functional additive. As compared to control chocolates, the supplementation with almost all freeze-dried fruit and vegetable powders caused significant (*p* < 0.05) decreases in the DPPH free radical scavenging activity in many types of obtained chocolates. Interestingly, among all studied chocolates, the highest DPPH radical scavenging capacity was exhibited by the DCH98S chocolate enriched with BEET powder. Nevertheless, the results indicated that the addition of BEET powder to DCH and DCH98ESt chocolates reduced DPPH radical scavenging activity significantly (*p* < 0.05) compared to the control. The observed differences in the free radical scavenging capacity of chocolates of different types with the same additive and different sweeteners may be due to different reaction mechanisms occurring during the preparation of the chocolates, including the Maillard reaction, the degradation of phenolic compounds of higher molecular weight to smaller phenolics and/or various transformations of flavan-3-ols and phenolic acids. In addition, other authors reported that supplementation of a sour cherry puree with sucrose or erythritol significantly declines its free radical scavenging activity [44]. They showed that the addition of natural sweeteners to a sour cherry puree resulted in a significant reduction in phenolic compounds, mainly flavan-3-ols. These phenomena may be due to the intermolecular interactions between the hydroxyl group from phenolic compounds in chocolate and phenolic-rich plant powders and a hydroxyl group in sucrose or sugar alcohol molecules [44,45].

It was observed that dark chocolates (98%) sweetened with erythritol and stevia instead of sucrose supplemented with RASB, BLCB and POME powders exhibited a higher DPPH radical-scavenging activity as compared to control DCH98ESt samples. Regarding the DCH chocolates, the enrichment with POME powder significantly increased (*p* < 0.05) the DPPH antioxidant capacity compared to the control sample. The obtained data highlighted, moreover, that the addition of freeze-dried berries, POME and BEET powders to MCH samples caused a significant (*p* < 0.05) increase in DPPH radical scavenging activity. As expected, the lowest free radical scavenging abilities was exhibited by the control milk chocolate. Our results also show that the partial sweetener substitution by BLCB powder caused the most pronounced reduction of the DPPH radical-scavenging potential of all dark chocolates. This result may be attributed to the synergistic and antagonistic interaction that results from the coexistence of many antioxidant compounds in enriched chocolates [41]. Some reports revelated that interactions between flavan-3-ols and anthocyanins might accelerate the degradation of anthocyanin pigments that further react giving rise to polymeric brown pigments [44]. It should be understood that the antioxidant activity of a mix is not the sum of the antioxidant activities of each of the components, due to interactions between the components. Therefore, it is difficult to predict in advance the result of food functionalization into components with antioxidant properties, e.g., the antioxidant potential of the product, inhibition of the growth of cancer cells or other biological properties. The effects can be surprising. Godočiková et al. (2017) observed that dark chocolates enriched with mulberry and sea buckthorn exhibited higher DPPH scavenging activity than control ones. Antioxidant capacities significantly increased with the addition of capsules of bioactive compounds [41].

The addition of freeze-dried phenolic-rich plant powders led to significant (*p* < 0.001) differences in the reducing capacity of all types of chocolates (Table S2). The ferric-reducing antioxidant power of the tested chocolate samples varied from 140.15 to 354.38 μmol TE/g DM (Figure 1II).

Among investigated samples, the DCH98ESt chocolates enriched with POME and BEET powders demonstrated the highest FRAP values. The obtained results indicated that the reducing capacity of dark chocolates made with sucrose noticeably decreases after supplementation of BLCB, POME and BEET powders. While in the case of dark chocolate sweetened with erythritol and stevia instead of sucrose, the addition of all functional powders led to a considerable increase in the reducing power compared to the control ones. This phenomenon may be ascribed to the interaction of phenolic compounds with sucrose. For example, Shalaby et al. (2016) showed that the introduction of sucrose to green tea significantly decreased its antioxidant potential [45]. In the present study, irrespective of the functional enrichment type, there was a significant increase in the ferric-reducing ability of all supplemented milk chocolates. Other authors have also observed that the addition of different sweeteners to fruit puree and green or black tea affects their antioxidant properties in different ways [44,45]. Therefore, we can conclude that the observed differences in DPPH and FRAP values of dark chocolates (98%) with the same powders and different sweeteners may be due to different reaction mechanisms occurring between phenolic compounds and sucrose and erythritol with stevia glycosides. Interestingly, unlike dark chocolates, the addition of BLCB powder caused the greatest increase in the reducing capacity of milk chocolate. Our results also showed that the addition of anthocyanin-rich BLUB powder caused an increase in reducing power regardless of the type of chocolate. This increase may be attributed to the fact that anthocyanins can act as reducing agents mainly through the electron-transfer mechanism [36].

Other studies have also reported that the addition of fruit or other phenolic-rich plants to chocolates either increase or decrease antioxidant activity evaluated by FRAP and DPPH assays [32,33,41,46]. These distinct differences may be attributed to the increased interaction between phenolics and other compounds, including carbohydrates, sweeteners, and proteins, present in chocolate and in fruits or vegetables. It is well known that both non-oxidized and oxidized phenolic compounds have a strong affinity to proteins, polysaccharides, alkaloids and Maillard reaction products, and may form insoluble complexes [46]. The results provide strong evidence that the interactions of mixtures of antioxidant compounds might generate synergic or inhibitor effects and can enhance or inhibit the antioxidant activity or even modify their reaction mechanisms [41].

### 2.4. Electronic Nose Analysis of Chocolates

Chocolate aroma depends on the combination of many volatile compounds (VCs) derived from cocoa beans and other ingredients, such as sucrose (sweetener), milk, and flavors, formed or modified during the roasting, alkalization and conching stages. It is well known that the typical chocolate flavor is mainly formed due to the Maillard reactions and the Strecker degradation of flavor precursors, such as free amino acids, short-chain peptides, and reducing sugars (e.g., glucose) during roasting [47,48]. However, it has to be noticed that functional additives rich in phenolic compounds having a positive effect on biological activity of enriched chocolates, can also affect the sensory properties of the final product. For example, phenolic compounds are responsible for the specific astringent and bitter taste of the raw beans and influence the stability and digestibility of the products obtained from them as a result of the formation of complexes mainly with polysaccharides, proteins, methylxanthines and Maillard reaction products. Therefore, these compounds are playing an important role in shaping the sensory characteristics of chocolates, fruits, and vegetables, as well as products obtained from them [48].

In our study, volatile compounds in chocolates were determined by using an electronic nose. A total of twenty-six VCs were identified, including alcohols, phenols, aldehydes, ketones, esters, acids, pyrazines, furfural, lactone, and sulfide compound in the all-enriched chocolates (Table 4). The results revealed that the addition of berries, POME and BEET powders significantly (*p* < 0.05) influenced the sensory attributes of the resultant chocolates. A two-way ANOVA revealed that the content of all VCs, apart from vanillin, varied significantly with chocolate type (*p* < 0.001), freeze-dried phenolic-rich plant powders (*p* < 0.001) and their interaction (*p* < 0.001) (Table S3, Supplementary Materials). The most important compounds of control and supplemented chocolates were acetic acid, benzaldehyde, 2-methylpropanal, 3-methylbutanal, 2-furfural, 2,5-dimethylpyrazine, pentanal and phenylethylacetate. The compound found in the highest concentration in all samples was acetic acid, which is associated with sour, pungent, and unpleasant notes. This compound is the highest odor-active compound in unroasted cocoa beans. Despite the fact that during further processing of cocoa beans acetic acid concentration decreases by over 70%, it is still the highest odor-active compound in roasted cocoa beans, cocoa mass and chocolates obtained from them [47,48,49,50]. The second compound found in the highest concentration in all tested chocolates is benzaldehyde. This compound has a pleasant fruity-type odor and a fruity-type flavor.

The presence of pyrazines, aldehydes and furfural was attributed to Maillard reactions. Among the aldehydes characteristic of the Strecker degradation, which is one of the main stages of the Maillard reaction, 3-methylbutanal, 2-methylpropanal and dimethyl disulfide derived from the decomposition of leucine, valine, and methionine, respectively, were determined. 3-Methylbutanal and 2-methylpropanal are very important compounds that have a positive effect on the development of the characteristic chocolate aroma of cocoa products [47,48,49,50].

**Figure 2:**
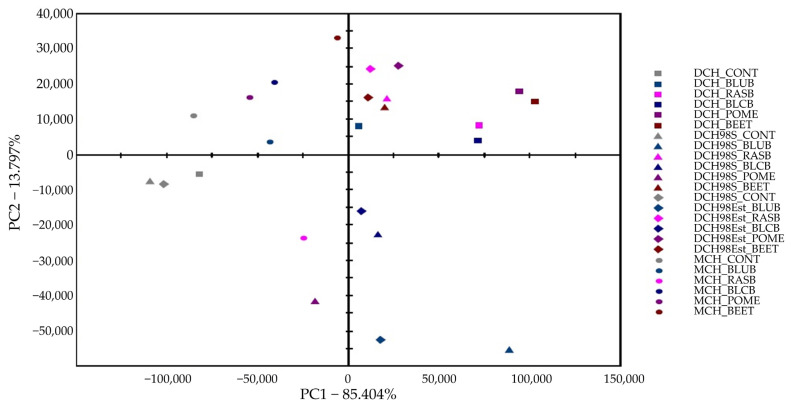
Principal component analysis (PCA) scores plot of aroma signals of different types of chocolate enriched with different freeze-dried phenolic-rich plant powders: DCH (**square**), DCH98S (**triangle**), DCH98ESt (**diamond**) and MCH (**dot**). Blueberries (BLUB—blue), raspberries (RASB—pink), blackberries (BLCB—dark blue), pomegranates pomace (POME—violet), beetroots (BEET—dark red), control (CONT—gray).

The principal component analysis (PCA) showed that dark and milk chocolates made with the addition of different functional additives are markedly different in terms of their VCs and thus clustered separately (Figure 2).

It is clear that each type of chocolate enriched with various functional additives was clearly distinguished by PC1 into two clusters, which suggested that the substitution of sweetener by 1% of fruit or vegetable powder resulted in the majority of the variance in the VCs composition compared to the corresponding control chocolate. It was demonstrated that almost all enriched chocolates could be distinguished from the control chocolates due to the abundance of some aldehydes, ketones, alcohols and acetic acid contents and the emergence of γ-nonalactone, which was not present in the control chocolates. In all chocolates enriched with berries, pomegranates pomace and beetroot powders the same classes of VCs were observed which were identified in control chocolates, in addition to γ-nonalactone. From the detected VCs, mainly 3-methylbutanal, phenylethylacetate, 2-phenylethanol, 2,5-dimethylpyrazine, 2,3-butanediol were positively correlated with chocolate aroma. However, the presence of benzaldehyde and pentanal with bitter and pungent notes origin from lipid oxidation was negatively correlated with chocolate flavor quality [47,48,49].

3-Methylbutanal produce key cocoa aromas such as malty and chocolate notes. Phenylethylacetate and 2-phenylethanol confer pleasant flowery and honey flavor notes enhancing flavor impression. 2,3-Butanediol, with the natural odor of cocoa butter, has been considered an important compound that could alter the overall aroma of chocolate [47,48,49].

Generally, the concentration of 2,3-pentanedione, γ-nonalactone and dimethyl trisulfide benzaldehyde, pentanal was significantly increased by the addition of fruit or vegetable powder. As demonstrated in Table 4, significant differences (*p* < 0.05) in the content of acetic acid were found between chocolates with different functional additives. The results showed that, depending on the functional ingredient type, a substantial change in the content of acetic acid was observed in all samples. Interestingly, all chocolates supplemented with RASB and BEET powder contained significantly lower amounts of acetic acid while those enriched with BLUB and POME powder had higher amounts of acetic acid than control chocolates. Independent of chocolate type, chocolates supplemented with BLUB and POME powders showed the highest content of acetic acid, while those enriched with RASB and BEET powders exhibited the highest content of benzaldehyde. On the other hand, the concentrations of 3-methylbutanal were higher in the control than in almost all enriched chocolates, mainly those made with BLUB or POME powder. Nevertheless, almost all of the enriched chocolates showed higher levels of alcohols, such as 2-phenylethanol and 2,3-butanediol, which are desirable to obtain cocoa products with flowery and honey aromas [49]. Thus, chocolates made with the addition of berries, pomegranates pomace and beetroot powders may have a good consumer acceptability when compared to control dark and milk chocolates.

## 3. Materials and Methods

### 3.1. Materials

The research materials were chocolates supplemented with lyophilized fruits and vegetables rich in flavonoids, including anthocyanins and phenolic acids. Chocolates were obtained from the following raw materials: Cocoa liquor (with 55% *w*/*w* of fat) and butter were purchased from Barry Callebaut (Łódź, Poland). Sugar, alkalized cocoa powder (with 10% *w*/*w* of fat), skimmed milk powder (with 1.5% *w*/*w* of fat), soy lecithin, polyglycerol polyricinoleate—PGPR emulsifier and ethyl vanillin were obtained from WIEPOL Zakład Pracy Chronionej Ireneusz Wielimborek (Sierpc, Poland). Erythritol with the addition of stevia (99% erythritol and 1% stevia) was purchased from Domos Polska Sp. z o.o. (Czosnów, Polska). Plants, such as blueberries (BLUB), raspberries (RASB), blackberries (BLCB), pomegranates (POME), and beetroots (BEET) were bought on the local market.

### 3.2. Chemicals and Reagents

Standards of catechin (Cat), epicatechin (ECat), procyanidin B2 (PC B2), procyanidin C1 (PC C1), gallic acid (GA), protocatechuic acid (PA), *p*-hydroxybenzoic acid (*p*-HBA), cyanidin-3-*O*-glucoside (Cy-3-Glu), 6-hydroxy-2,5,7,8-tetramethylchroman-2-carboxylic acid (Trolox), 2,2′-azino-bis (3-ethylbenzothiazoline-6-sulfonic acid) diammonium salt (ABTS), 2,2-diphenyl-1-(2,4,6-trinitrophenyl) hydrazyl (DPPH), 2,4,6-tri(2-pyridyl)-s-triazine (TPTZ), sodium acetate, ferric chloride hexahydrate, ferrozine, and ammonium acetate were purchased from Sigma-Aldrich (St. Louis, MO, USA). HPLC grade methyl tert-butyl ether (MTBE) and methanol were purchased from J.T. Baker (Deventer, The Netherlands). All other reagents were of analytical grade and were purchased from Chempur (Piekary Śląskie, Poland). Chromacol PTFE syringe filters (0.2 µm pore size) were purchased from Shim-Pol (Izabelin, Poland). Ultrapure water (resistivity 18.2 MΩ cm), obtained from a Milli-Q purification system (Millipore, Bedford, MA, USA), was used for all analyses.

### 3.3. Lyophilization of Fruits and Vegetables

Plant materials, i.e., pomegranates pomace, whole berries and beetroots cut into cubes, were frozen at −80 °C for 48 h. Then they were freeze-dried in a BETTA2-8LSC plus Christ freeze drier (Osterode am Harz, Germany) for 24 h. The initial parameters of the process were—pressure: 1 millibar, shelf temperature: 5 °C, while final parameters—pressure: 0.1 millibar, shelf temperature: 5 °C. The obtained freeze-dried products were then ground to a fine powder in an XB-9103 MPM PRODUCT knife mill (Milanówek, Poland) and stored in glass containers.

### 3.4. Preparation of Chocolates

Four types of chocolate with 1% addition of fruit or vegetable powders were prepared during the study: dark chocolate 53% (~53% cocoa) with the total fat content of 35% (*w*/*w*) and sweetened with sucrose (DCH), dark chocolate 98% (~98% cocoa) with the total fat content of 51% (*w*/*w*) sweetened with sucrose (DCH98S), dark chocolate 98% (~98% cocoa) with the total fat content of 51% (*w*/*w*) sweetened with erythritol with stevia (DCH98ESt), milk chocolate (~40% cocoa, 20% skimmed milk powder) with the total fat content of 36% (*w*/*w*) and sweetened with sucrose (MCH). Recipes of chocolates are given in Table 5.

Furthermore, control chocolates were obtained in which an additional portion of the sweetener was added to the chocolates instead of the freeze-dried fruits or vegetables. For example, in the DCH, the sucrose percentage was then 45.59% instead of 44.59%.

The preparation process of chocolates consisted of the following basic stages: grinding in the ball mill type M-5 (Promet, Łódź, Poland; loading up to 6 kg), conching in the K-5 type conch (Promet, Łódź, Poland; loading up to 6 kg), tempering in the temperer model T8 type Temperatrice containing a closed cooling system with an additional AC current heat exchanger (Pomati Group Srl, Zona Ind. Mirandolina, Italy; loading up to 8 kg), moulding, cooling and wrapping.

The cocoa butter, liquor and eventually cocoa powder were liquefied in the preheated to 70 °C ball mill. Next, sugar, milk powder (in the case of MCH) and 50% of the amount of lecithin were dosed into the ball mill. The grinding was carried out at 70 °C for 50 min (DCH), at 85 °C for 90 min (DCH98S and DCH98ESt) or at 55 °C for 60 min (MCH) with a rotational velocity of 75 rpm to obtain a particle size in the range of 20–25 μm, which was measured using the micrometric screw NSK Digitrix-MARK II ELECTRONIC MICROMETER with the electronic readout of the results (Japan Micrometer MFG. Co., Ltd., Tokyo, Japan). After this time, the obtained cocoa mass was transferred to preheated to 50 °C conch for further homogenization and emulsification. Then, freeze-dried phenolic-rich plant powders were introduced into the conch. After 45 min of conching, ethyl vanillin, PGPR emulsifier and the remaining part of the lecithin were added to the mass, and then conching was continued for 15 min. Next, the mass was subjected to tempering. For this purpose, the temperature of the mass was lowered in the temperer from 50 to 32 °C in the case of dark chocolates and to 28 °C in the case of milk chocolate. These temperatures were maintained for 15 min. Finally, chocolate masses were poured to preheated to 30 °C (dark chocolates) or 27 °C (milk chocolate) forms, cooled to 18 °C in a cooling tunnel (Promet, Łódź, Poland), and removed from the forms. Chocolate bars were wrapped in aluminum foil and subjected to analysis. All chocolates were obtained in triplicate.

### 3.5. Water Content and Water Activity Determination

Water content was determined by drying the ground chocolate samples mixed with sand at 102–105 °C to constant weight [28].

The water activity of the chocolates was determined by using HYGROPALM AW1 meter (Rotronic, Helvetia, Switzerland) equipped with a digital probe AW-DIO [28].

### 3.6. Color Determination

The color was determined using a trichromatic reflection colorimeter Konica Minolta CR-400 with Spectra Magic NX 1.3 software (Konica Minolta Sensing INC., Osaka, Japan). The results were expressed in accordance with CIE L*a*b* system (D65 illuminant and 10° viewing angle) [51].

### 3.7. UHPLC-DAD-ESI–MS/MS Analysis of Phenolic Compounds

The phenolic compounds were extracted according to the method described by Żyżelewicz et al. (2018), with some modifications [28]. Briefly, the accurately weighed defatted chocolate samples were extracted 3 times in an orbital shaker at room temperature for 30 min at 150× *g* with a mixture of acetone/water/acetic acid (70/29.5/0.5, *v*/*v*/*v*). The mixture was centrifuged at 4000× *g* for 10 min and the supernatant from each extraction were combined and evaporated under a stream of nitrogen. The residues were dissolved in methanol and filtered through a 0.20 μm pore size PTFE syringe filters. Finally, the samples were analyzed for the content of phenolic compounds using a UHPLC+ Dionex UltiMate 3000 system equipped with a UV–Vis diode array detector (Thermo Fisher Scientific Inc., Waltham, MA, USA), and a Transcend™ TLX-2 multiplexed LC system equipped with Q-Exactive Orbitrap mass spectrometer (Thermo Scientific, Hudson, NH, USA) using a heated electrospray ionization (ESI) interface (HESI–II). Samples (10 μL) were injected on an Accucore™ C18 column (150 mm × 2.1 mm i.d., 2.6 µm; Thermo Fisher Scientific Inc., Waltham, MA, USA). The column temperature was set at 30 °C. The mobile phase and gradient program were used as previously described by Oracz, Nebesny and Żyżelewicz (2019), with some modifications [52]. The 2-phase solvent system used for phenolic compounds separation was composed of 0.1% formic acid in water as solvent A and 0.1% formic acid in acetonitrile as solvent B. The flow rate was 0.35 mL/min and the gradient was as follows: 0–8 min, 1–5% B; 8–15 min, 5–8% B; 15–20 min, 8–10% B; 20–25 min, 10–15% B; 25–35 min, 15–20% B; 35–40 min, 20–25% B; 40–50 min, 25–90% B; 50–53 min, 90% B; 53–58 min, 90–1% B. Finally, the initial conditions were held for 7 min for column re-equilibration and for 5 min as a re-equilibration step. UV–Vis detection was performed at 280 nm for flavan-3-ols and phenolic acids and at 520 nm for anthocyanins. Instrument control, data acquisition, and evaluation were conducted with Chromeleon 6.8 Chromatography Data System, Qexactive Tune 2.1, Aria 1.3.6, and Thermo Xcalibur 2.2 software, respectively. Phenolic compounds were identified by comparing their retention times, UV–Vis absorbance spectra, full scan mass spectra, and MS/MS fragmentation patterns with their corresponding standards analyzed under identical conditions and previous literature reports [53,54]. Quantification was carried out using an external standard method. The concentration of individual flavan-3-ols and phenolic acids was determined based on peak area and calibration curves derived from corresponding reference compounds. For the quantification of anthocyanins, the calibration curves of Cy-3-Glu were used. All measurements were conducted in triplicate and results were expressed as mg phenolic compound per 100 g chocolate dry mass (mg/100 g DM).

### 3.8. Free Radical Scavenging Assay

The free radical scavenging activity was determined using the DPPH assay [28]. The analytical samples were prepared using serial dilutions of chocolate extracts in methanol. For each sample, experiments were conducted in triplicate. Finally, the mean concentration of the test chocolate extracts at which the concentration of the DPPH free radicals was reduced by 50% (IC_50_) was calculated.

### 3.9. Ferric Reducing Antioxidant Power Assay

The ferric reducing ability (FRAP) was evaluated using the method of Oracz and Żyżelewicz (2019) [55]. For each sample, experiments were conducted in triplicate. The results were expressed as µmol Trolox equivalents per gram of chocolate DM (μmol TE/g DM).

### 3.10. Electronic Nose Analysis of Tested Chocolates

Electronic nose (E-nose) analysis of volatile flavor compounds was carried out according to the method of Rottiers et al. (2019), with some modifications [50]. The E-nose analyses were performed using a commercial Heracles II electronic nose (Alpha MOS, Toulouse, France), equipped with an HS-100 autosampler, a sensor array unit, and 2 columns working in parallel mode: a non-polar column (MXT5: 5% diphenyl, 95% methylpolysiloxane, 10 m length and 180 lm diameter) and a slightly polar column (MXT1701: 14% cyanopropylphenyl, 86% methylpolysiloxane, 10 m length and 180 lm diameter). An accurately weighed 1.0 g chocolate sample was put into 20-mL screw vials sealed with a magnetic cap with polytetrafluorethylene-silicone septa and placed in the auto-sampler. The vials were incubated in a shaker oven for 20 min at 50 °C and shaken at 500 rpm. Next, a syringe sampled 1000 µL of the headspace and then injected it into the gas chromatograph with 2 flame ionization detectors. The thermal program started at 50 °C (held for 2 s) and increased up to 250 °C at 3 °C/s and held for 21 s. The total separation time was 100 s. The calibration of the apparatus was carried out using a solution of alkanes (from *n*-hexane to *n*-hexadecane). The retention times of *n*-alkanes were used to determine the Kovats indices and identify the volatile compounds using AromaChemBase software (Alpha MOS, Toulouse, France). Each sample was measured in triplicate. Instrument control, data acquisition, and evaluation were conducted with Alphasoft 14.2 and AroChembase (Alpha MOS, Toulouse, France) softwares. The principal component analysis (PCA) was performed using AlphaSoft software (Alpha MOS, Toulouse, France) to determine the dissimilarities among the same types of chocolates in terms of volatile components.

### 3.11. Organoleptic Evaluation of Chocolates

The organoleptic evaluation of chocolates was carried out according to Żyżelewicz et al. (2018) [28] in our specialist sensory analysis laboratory. The evaluation was made by ten panelists using a 5-point scale with the relevant significance coefficients, in which 5 points corresponded to the best quality and 1 point to the worst. The sensory attributes of the chocolates, i.e., appearance in the packet, shape, color, consistency (hardness, smoothness), conchoidal fracture, aroma, taste, and upper and lower surface glossiness were evaluated. Final assessments were presented on a 5-point scale, according to which 5 meant extremely desirable quality, 4 was desirable quality, 3 was tolerable quality, 2 represented dislike, and 1 was for a defective product.

### 3.12. Statistical Analysis

The results are presented as mean ± standard deviations of 3 replicates. The one-way analysis of variance (ANOVA) was used to determine if there were significant differences between the physicochemical properties, phenolic compounds and antioxidant activity observed in the control and enriched chocolate samples. Where effects of supplementation of anthocyanin-rich were significant, the means were compared with Tukey’s HSD (Honestly Significant Difference) at *p* < 0.05, using the Statistica 13.0 software (StatSoft, Inc., Tulsa, OK, USA). The effects of the types of chocolates and different freeze-dried phenolic-rich plant powders and their interaction on phenolic content, antioxidant activity and volatile compounds content in chocolates were tested by means of two-way ANOVA.

## 4. Conclusions

The results of the present study revealed that the enrichment of dark and milk chocolates with berries, pomegranates pomace and beetroot powders caused an increase in the amount of phenolic compounds, including flavan-3-ols, anthocyanins and phenolic acids. Chocolates enriched with BLUB powder were characterized by the highest total polyphenol content. Our findings were further supported by the enhanced free radical scavenging activity and reducing capacity of different types of chocolates supplemented with fruit or vegetable powders. All chocolates with the addition of BLUB and RASB powders obtained an extremely desirable assessment in the organoleptic evaluation. The analysis of aroma compounds (volatile compounds) with the use of an electronic nose showed that berries, pomegranates pomace and beetroot powders have been successfully added to produce both dark and milk chocolates with acceptable sensory quality.

The physicochemical and sensory analysis results indicated that up to 1% of tested freeze-dried phenolic-rich plant powders can be successfully added to produce milk and dark chocolates with increased contents of polyphenols and good sensory properties.

## Figures and Tables

**Table 1:** Physicochemical characteristic and organoleptic assessment of different types of chocolates enriched with various freeze-dried phenolic-rich plant powders.

Chocolate Type	Functional Enrichment	Water Content (%)	Water Activity	CIE L*a*b* Color Parameters			Organoleptic Assessment (Point)
				L*	a*	b*	
DCH	CONT	1.15 ± 0.04 ^b^	0.408 ± 0.003 ^b^	27.79 ± 0.12 ^a^	5.30 ± 0.10 ^a^	1.15 ± 0.09 ^b^	4.8 ± 0.2 ^a^
	BLUB	1.63 ± 0.02 ^c^	0.426 ± 0.002 ^c^	29.88 ± 0.11 ^e^	5.33 ± 0.09 ^b^	1.63 ± 0.02 ^c^	4.9 ± 0.1 ^a^
	RASB	2.19 ± 0.09 ^f^	0.446 ± 0.005 ^d^	29.17 ± 0.11 ^b^	5.54 ± 0.05 ^d^	2.19 ± 0.06 ^f^	4.8 ± 0.2 ^a^
	BLCB	1.73 ± 0.08 ^d^	0.490 ± 0.002 ^f^	29.78 ± 0.12 ^d^	5.39 ± 0.09 ^c^	1.73 ± 0.02 ^d^	4.8 ± 0.2 ^a^
	POME	1.83 ± 0.05 ^e^	0.400 ± 0.007 ^a^	29.58 ± 0.13 ^c^	5.50 ± 0.11 ^d^	1.83 ± 0.03 ^e^	4.7 ± 0.2 ^a^
	BEET	0.93 ± 0.04 ^a^	0.455 ± 0.003 ^e^	31.69 ± 0.10 ^f^	5.39 ± 0.08 ^c^	0.93 ± 0.04 ^a^	4.3 ± 0.3 ^b^
DCH98S	CONT	1.63 ± 0.02 ^b^	0.409 ± 0.001 ^b^	27.70 ± 0.11 ^b^	3.75 ± 0.09 ^e^	1.63 ± 0.08 ^b^	4.5 ± 0.1 ^a^
	BLUB	1.97 ± 0.01 ^f^	0.378 ± 0.002 ^a^	27.74 ± 0.12 ^b^	3.51 ± 0.10 ^c^	1.97 ± 0.07 ^f^	4.6 ± 0.1 ^a^
	RASB	1.69 ± 0.04 ^c^	0.447 ± 0.003 ^d^	28.79 ± 0.14 ^e^	3.34 ± 0.12 ^a^	1.69 ± 0.02 ^c^	4.6 ± 0.2 ^a^
	BLCB	1.74 ± 0.07 ^d^	0.433 ± 0.006 ^c^	27.82 ± 0.11 ^c^	3.60 ± 0.03 ^d^	1.74 ± 0.06 ^d^	4.5 ± 0.2 ^a^
	POME	1.26 ± 0.02 ^a^	0.408 ± 0.003 ^b^	27.98 ± 0.13 ^d^	3.53 ± 0.07 ^c^	1.26 ± 0.04 ^a^	4.5 ± 0.2 ^a^
	BEET	1.86 ± 0.08 ^e^	0.445 ± 0.005 ^d^	27.57 ± 0.12 ^a^	3.40 ± 0.09 ^b^	1.86 ± 0.02 ^e^	4.1 ± 0.3 ^b^
DCH98ESt	CONT	1.83 ± 0.05 ^c^	0.408 ± 0.002 ^b^	27.22 ± 0.10 ^a^	3.36 ± 0.07 ^e^	1.83 ± 0.03 ^c^	4.4 ± 0.2 ^a^
	BLUB	1.58 ± 0.04 ^a^	0.394 ± 0.003 ^a^	27.96 ± 0.10 ^c^	3.25 ± 0.10 ^d^	1.58 ± 0.02 ^a^	4.5 ± 0.1 ^a^
	RASB	1.60 ± 0.02 ^a^	0.443 ± 0.001 ^d^	28.78 ± 0.12 ^e^	3.11 ± 0.09 ^b^	1.60 ± 0.01 ^a^	4.5 ± 0.2 ^a^
	BLCB	2.03 ± 0.03 ^d^	0.445 ± 0.002 ^d^	29.31 ± 0.11 ^f^	3.03 ± 0.05 ^a^	2.03 ± 0.06 ^d^	4.4 ± 0.2 ^a,b^
	POME	1.81 ± 0.01 ^c^	0.392 ± 0.004 ^a^	27.85 ± 0.09 ^b^	3.49 ± 0.07 ^f^	1.81 ± 0.03 ^c^	4.3 ± 0.1 ^a,b^
	BEET	1.70 ± 0.04 ^b^	0.436 ± 0.003 ^c^	28.35 ± 0.13 ^d^	3.18 ± 0.09 ^c^	1.70 ± 0.02 ^b^	3.9 ± 0.3 ^c^
MCH	CONT	1.22 ± 0.06 ^c^	0.449 ± 0.004 ^b^	32.99 ± 0.10 ^b^	7.54 ± 0.06 ^b^	1.22 ± 0.05 ^b^	4.8 ± 0.2 ^a^
	BLUB	1.24 ± 0.03 ^c^	0.393 ± 0.003 ^a^	34.01 ± 0.15 ^f^	7.25 ± 0.08 ^a^	1.24 ± 0.03 ^c^	4.9 ± 0.1 ^a^
	RASB	1.17 ± 0.04 ^b^	0.441 ± 0.002 ^b^	33.16 ± 0.12 ^d^	7.60 ± 0.11 ^b^	1.17 ± 0.02 ^b^	4.8 ± 0.2 ^a^
	BLCB	1.19 ± 0.05 ^b^	0.475 ± 0.001 ^c^	32.29 ± 0.11 ^a^	7.55 ± 0.06 ^b^	1.19 ± 0.05 ^b^	4.8 ± 0.2 ^a^
	POME	0.94 ± 0.04 ^a^	0.396 ± 0.002 ^a^	33.37 ± 0.11 ^e^	7.99 ± 0.07 ^d^	0.94 ± 0.02 ^a^	4.6 ± 0.1 ^b^
	BEET	1.24 ± 0.07 ^c^	0.446 ± 0.003 ^b^	33.08 ± 0.12 ^c^	7.70 ± 0.09 ^c^	1.24 ± 0.08 ^c^	3.8 ± 0.3 ^c^

Blueberries (BLUB), raspberries (RASB), blackberries (BLCB), pomegranates pomace (POME), beetroots (BEET), control chocolate, (CONT). Data are presented as mean ± SD of three replications. The values followed by the same lowercase letter (a–f) within each chocolate type in the same column do not differ significantly according to Tukey’s HSD test at *p* < 0.05.

**Table 2:** Phenolic compounds content and antioxidant properties of studied freeze-dried phenolic-rich plant powders.

Compounds and Antioxidant Activity	BLUB	RASB	BLCB	POME	BEET
**Phenolic Content**					
*Flavan-3-ols (mg/100 g DM)*					
Cat	35.41 ± 0.12 ^e^	30.70 ± 0.14 ^d^	10.09 ± 0.09 ^a^	11.56 ± 0.11 ^b^	21.87 ± 0.15 ^c^
Ecat	57.45 ± 0.24 ^d^	52.23 ± 0.19 ^b^	48.34 ± 0.18 ^a^	55.34 ± 0.26 ^c^	58.50 ± 0.31 ^e^
PC B2	39.34 ± 0.17 ^d^	20.46 ± 0.19 ^c^	43.24 ± 0.21 ^e^	16.78 ± 0.13 ^b^	13.65 ± 0.10 ^a^
PC C1	11.98 ± 0.08 ^e^	0.06 ± 0.02 ^a^	0.12 ± 0.03 ^c^	0.09 ± 0.02 ^b^	0.05 ± 0.02 ^a^
Total	144.18 ± 0.56 ^e^	103.45 ± 0.25 ^d^	101.79 ± 0.21 ^c^	83.77 ± 0.14 ^a^	94.07 ± 0.18 ^b^
*Anthocyanins (mg/100 g DM)*					
Cy-3-Glu	nd	3730.65 ± 10.87 ^a^	4370.09 ± 12.44 ^b^	nd	nd
Cy-3-Rut	nd	nd	59.76 ± 0.21	nd	nd
Cy-3,5-diGlu	10,280.60 ± 32.51 ^b^	nd	nd	98.69 ± 0.36 ^a^	nd
Cy-3-Xyl	4195.67 ± 14.41 ^b^	nd	164.86 ± 0.41 ^a^	nd	nd
Cy-3-(6″-Mal-Glu)	nd	nd	315.46 ± 0.65 ^b^	nd	nd
Del-3,5-diGlu	7697.48 ± 26.54 ^b^	nd	nd	31.56 ± 0.21 ^a^	nd
Del-3-Glu	nd	nd	nd	8.49 ± 0.07	nd
Pel-3,5-diGlu	10,759.88 ± 36.38	nd	nd	nd	nd
Total	32,933.63 ± 61.89 ^d^	3730.65 ± 10.87 ^b^	4910.17 ± 13.70 ^c^	138.74 ± 0.39 ^a^	nd
*Phenolic Acids (mg/100 g DM)*					
GA	9.36 ± 0.09 ^b^	nd	2.57 ± 0.05 ^a^	nd	nd
PA	6.14 ± 0.05 ^c^	3.19 ± 0.05 ^b^	2.24 ± 0.04 ^a^	nd	26.48 ± 0.11 ^d^
*p*-HBA	1.83 ± 0.03 ^a^	10.29 ± 0.08 ^b^	18.48 ± 0.12 ^d^	11.80 ± 0.06 ^c^	70.40 ± 0.19 ^e^
Total	17.33 ± 0.25 ^c^	13.48 ± 0.08 ^b^	23.29 ± 0.22 ^d^	11.80 ± 0.05 ^a^	96.88 ± 0.36 ^e^
Total phenolics (mg/100 g DM)	33,095.14 ± 62.70 ^e^	3847.58 ± 11.20 ^c^	5035.25 ± 14.13 ^d^	234.31 ± 0.58 ^b^	190.95 ± 0.54 ^a^
***Antioxidant Activity***					
DPPH EC_50_ (mg/mg DPPH)	0.15 ± 0.02 ^a^	0.62 ± 0.03 ^b^	1.17 ± 0.05 ^c^	1.49 ± 0.04 ^d^	0.15 ± 0.02 ^a^
FRAP (μmol TE/g DM)	761.27 ± 0.13 ^e^	646.86 ± 0.16 ^c^	629.61 ± 0.09 ^b^	671.25 ± 0.12 ^d^	498.34 ± 0.18 ^a^

Blueberries (BLUB), raspberries (RASB), blackberries (BLCB), pomegranates pomace (POME), beetroots (BEET), control chocolate, (CONT). Data are presented as mean ± SD of three replications. The values followed by the same lowercase letter (a–e) in the same row do not differ significantly according to Tukey’s HSD test at *p* < 0.05. nd—not detected.

**Table 3:** The content of individual phenolic compounds in different types of chocolates enriched with various freeze-dried phenolic-rich plant powders (mg/100 g DM).

Phenolic Compounds	Functional Enrichment					
	CONT	BLUB	RASB	BLCB	POME	BEET
**DCH**						
***Flavan-3-ols***						
Cat	14.15 ± 0.09 ^a^	18.66 ± 0.08 ^e^	16.60 ± 0.07 ^d^	14.18 ± 0.11 ^b^	15.16 ± 0.12 ^c^	17.89 ± 0.09 ^e^
Ecat	77.23 ± 0.48 ^a^	97.32 ± 0.43 ^f^	85.24 ± 0.47 ^c^	80.68 ± 0.38 ^b^	88.67 ± 0.34 ^d^	96.54 ± 0.51 ^e^
PC B2	23.30 ± 0.11 ^a^	43.19 ± 0.15 ^d^	43.35 ± 0.16 ^d^	45.49 ± 0.12 ^e^	40.41 ± 0.18 ^c^	34.89 ± 0.15 ^b^
PC C1	10.47 ± 0.06 ^a^	17.79 ± 0.07 ^e^	11.80 ± 0.08 ^b^	14.71 ± 0.09 ^d^	11.86 ± 0.07 ^b^	14.40 ± 0.06 ^c^
Total flavan-3-ols	125.15 ± 0.54 ^a^	176.96 ± 0.59 ^d^	156.99 ± 0.66 ^b^	156.06 ± 0.65 ^b^	156.10 ± 0.58 ^b^	163.72 ± 0.63 ^c^
***Anthocyanins***						
Cy-3-Glu	nd	nd	3.73 ± 0.05 ^a^	4.37 ± 0.04 ^b^	nd	nd
Cy-3-Rut	nd	nd	nd	0.06 ± 0.01 ^a^	nd	nd
Cy-3,5-diGlu	nd	10.28 ± 0.15 ^b^	nd	nd	0.10 ± 0.04 ^a^	nd
Cy-3-Xyl	nd	4.20 ± 0.07 ^b^	nd	0.16 ± 0.07 ^a^	nd	nd
Cy-3-(6″-Mal-Glu)	nd	nd	nd	0.32 ± 0.03	nd	nd
Del-3,5-diGlu	nd	7.70 ± 0.10 ^b^	nd	nd	0.03 ± 0.05 ^a^	nd
Del-3-Glu	nd	nd	nd	nd	0.01 ± 0.01	nd
Pel-3,5-diGlu	nd	10.76 ± 0.09	nd	nd	nd	nd
Total anthocyanins	nd	32.93 ± 0.31 ^d^	3.73 ± 0.05 ^b^	4.91 ± 0.15 ^c^	0.14 ± 0.09 ^a^	nd
***Phenolic acids***						
GA	7.23 ± 0.09 ^b^	8.77 ± 0.11 ^c^	7.42 ± 0.10 ^b^	7.12 ± 0.12 ^a^	9.25 ± 0.10 ^d^	8.88 ± 0.11 ^c^
PA	1.34 ± 0.04 ^a^	4.24 ± 0.10 ^e^	2.86 ± 0.07 ^c^	2.41 ± 0.06 ^b^	2.91 ± 0.08 ^c^	3.07 ± 0.05 ^d^
*p*-HBA	7.69 ± 0.09 ^a^	8.25 ± 0.11 ^c^	8.60 ± 0.05 ^d^	7.90 ± 0.09 ^b^	7.96 ± 0.10 ^b^	8.90 ± 0.03 ^e^
Total phenolic acids	16.26 ± 0.21 ^a^	21.26 ± 0.24 ^f^	18.88 ± 0.21 ^c^	17.43 ± 0.18 ^b^	20.12 ± 0.26 ^d^	20.85 ± 0.19 ^e^
**Total phenolics**	**141.41 ± 0.74 ^a^**	**231.16 ± 0.63 ^d^**	**179.60 ± 0.64 ^b^**	**177.40 ± 0.70 ^b^**	**176.36 ± 0.71 ^b^**	**184.57 ± 0.84 ^e^**
**DCH98S**						
***Flavan-3-ols***						
Cat	28.48 ± 0.10 ^c^	31.73 ± 0.08 ^d^	28.21 ± 0.14 ^c^	23.14 ± 0.12 ^a^	25.77 ± 0.09 ^b^	33.00 ± 0.14 ^e^
Ecat	152.29 ± 0.51 ^a^	165.44 ± 0.45 ^e^	164.91 ± 0.31 ^d^	154.30 ± 0.39 ^b^	155.17 ± 0.43 ^c^	173.70 ± 0.23 ^f^
PC B2	39.61 ± 0.12 ^a^	63.42 ± 0.11 ^e^	53.69 ± 0.14 ^c^	48.43 ± 0.09 ^b^	70.96 ± 0.10 ^f^	59.39 ± 0.19 ^d^
PC C1	21.20 ± 0.08 ^b^	29.25 ± 0.10 ^e^	20.05 ± 0.06 ^a^	27.95 ± 0.07 ^c^	21.23 ± 0.09 ^b^	28.73 ± 0.11 ^d^
Total flavan-3-ols	241.58 ± 0.78 ^a^	289.84 ± 0.69 ^e^	266.86 ± 0.58 ^c^	253.82 ± 0.72 ^b^	273.13 ± 0.73 ^d^	294.82 ± 0.63 ^f^
***Anthocyanins***						
Cy-3-Glu	nd	nd	3.73 ± 0.06 ^a^	4.37 ± 0.05 ^b^	nd	nd
Cy-3-Rut	nd	nd	nd	0.06 ± 0.02	nd	nd
Cy-3,5-diGlu	nd	10.28 ± 0.11 ^b^	nd	nd	0.10 ± 0.02 ^a^	nd
Cy-3-Xyl	nd	4.20 ± 0.08 ^b^	nd	0.16 ± 0.04 ^a^	nd	nd
Cy-3-(6″-Mal-Glu)	nd	nd	nd	0.32 ± 0.03	nd	nd
Del-3,5-diGlu	nd	7.70 ± 0.12 ^b^	nd	nd	0.03 ± 0.01 ^a^	nd
Del-3-Glu	nd	nd	nd	nd	0.01 ± 0.01	nd
Pel-3,5-diGlu	nd	10.76 ± 0.17	nd	nd	nd	nd
Total anthocyanins	nd	32.94 ± 0.34 ^d^	3.73 ± 0.06 ^b^	4.91 ± 0.14 ^c^	0.14 ± 0.04 ^a^	nd
***Phenolic acids***						
GA	11.29 ± 0.11 ^a^	14.91 ± 0.09 ^e^	12.61 ± 0.10 ^b^	13.53 ± 0.12 ^d^	16.28 ± 0.11 ^f^	12.95 ± 0.08 ^c^
PA	2.58 ± 0.04 ^a^	7.21 ± 0.06 ^f^	4.86 ± 0.05 ^d^	4.59 ± 0.06 ^c^	5.20 ± 0.07 ^e^	3.72 ± 0.05 ^b^
*p*-HBA	9.78 ± 0.10 ^b^	14.02 ± 0.12 ^f^	10.41 ± 0.08 ^c^	11.21 ± 0.11 ^d^	13.53 ± 0.10 ^e^	8.59 ± 0.07 ^a^
Total phenolic acids	23.65 ± 0.24 ^a^	36.14 ± 0.27 ^f^	27.88 ± 0.23 ^c^	29.33 ± 0.28 ^d^	35.01 ± 0.28 ^e^	25.26 ± 0.20 ^b^
**Total phenolics**	**265.23 ± 1.02 ^a^**	**358.92 ± 1.32 ^f^**	**298.47 ± 0.87 ^c^**	**288.06 ± 0.94 ^b^**	**308.28 ± 0.95 ^d^**	**320.08 ± 0.82 ^e^**
**DCH98Est**						
***Flavan-3-ols***						
Cat	27.73 ± 0.13 ^d^	26.90 ± 0.12 ^c^	25.92 ± 0.14 ^b^	25.62 ± 0.11 ^a^	25.62 ± 0.13 ^a^	27.97 ± 0.11 ^e^
Ecat	149.37 ± 0.49 ^d^	140.24 ± 0.40 ^b^	139.79 ± 0.35 ^a^	139.84 ± 0.47 ^a^	141.54 ± 0.21 ^b^	147.25 ± 0.31 ^c^
PC B2	33.18 ± 0.17 ^a^	53.76 ± 0.16 ^e^	45.52 ± 0.15 ^c^	41.05 ± 0.14 ^b^	60.15 ± 0.21 ^f^	50.35 ± 0.16 ^d^
PC C1	17.76 ± 0.15 ^b^	24.80 ± 0.11 ^e^	17.00 ± 0.10 ^a^	23.70 ± 0.12 ^d^	18.00 ± 0.13 ^c^	24.35 ± 0.10 ^d^
Total flavan-3-ols	228.04 ± 0.66 ^a^	245.70 ± 0.55 ^c^	228.23 ± 0.61 ^a^	230.21 ± 0.59 ^b^	245.31 ± 0.69 ^c^	249.92 ± 0.53 ^d^
***Anthocyanins***						
Cy-3-Glu	nd	nd	3.74 ± 0.05 ^a^	4.38 ± 0.04 ^b^	nd	nd
Cy-3-Rut	nd	nd	nd	0.06 ± 0.02	nd	nd
Cy-3,5-diGlu	nd	10.30 ± 0.12 ^b^	nd	nd	0.10 ± 0.01 ^a^	nd
Cy-3-Xyl	nd	4.20 ± 0.09 ^b^	nd	0.17 ± 0.03 ^a^	nd	nd
Cy-3-(6″-Mal-Glu)	nd	nd	nd	0.32 ± 0.04	nd	nd
Del-3,5-diGlu	nd	7.71 ± 0.11 ^b^	nd	nd	0.03 ± 0.01 ^a^	nd
Del-3-Glu	nd	nd	nd	nd	0.01 ± 0.01	nd
Pel-3,5-diGlu	nd	10.78 ± 0.09	nd	nd	nd	nd
Total anthocyanins	nd	32.99 ± 0.35 ^d^	3.74 ± 0.05 ^b^	4.93 ± 0.13 ^c^	0.14 ± 0.03 ^a^	nd
*****Phenolic acids*****						
GA	11.14 ± 0.08 ^c^	12.64 ± 0.10 ^e^	10.69 ± 0.09 ^a^	11.47 ± 0.11 ^d^	13.80 ± 0.09 ^f^	10.98 ± 0.09 ^b^
PA	2.16 ± 0.04 ^a^	6.11 ± 0.03 ^f^	4.12 ± 0.05 ^d^	3.89 ± 0.06 ^c^	4.41 ± 0.07 ^e^	3.15 ± 0.05 ^b^
*p*-HBA	10.70 ± 0.09 ^c^	11.89 ± 0.10 ^f^	11.74 ± 0.06 ^e^	9.50 ± 0.08 ^b^	11.47 ± 0.11 ^d^	7.29 ± 0.06 ^a^
Total phenolic acids	24.00 ± 0.21 ^b^	30.64 ± 0.23 ^f^	26.55 ± 0.22 ^d^	24.86 ± 0.24 ^c^	29.68 ± 0.19 ^e^	21.42 ± 0.20 ^a^
**Total phenolics**	**252.04 ± 0.86 ^a^**	**309.33 ± 0.83 ^e^**	**258.52 ± 0.72 ^b^**	**260.00 ± 0.81 ^b^**	**275.13 ± 0.90 ^d^**	**271.34 ± 0.73 ^c^**
**MCH**						
***Flavan-3-ols***						
Cat	4.44 ± 0.03 ^a^	5.54 ± 0.04 ^b^	11.83 ± 0.03 ^d^	11.35 ± 0.04 ^c^	12.01 ± 0.05 ^e^	14.65 ± 0.11 ^f^
Ecat	45.58 ± 0.16 ^a^	60.00 ± 0.17 ^e^	58.72 ± 0.18 ^d^	56.92 ± 0.18 ^b^	58.21 ± 0.21 ^c^	64.64 ± 0.16 ^f^
PC B2	23.41 ± 0.12 ^a^	31.59 ± 0.11 ^e^	31.03 ± 0.10 ^d^	27.55 ± 0.13 ^b^	28.40 ± 0.11 ^c^	34.05 ± 0.14 ^f^
PC C1	6.23 ± 0.05 ^c^	18.90 ± 0.04 ^f^	5.90 ± 0.07 ^b^	7.36 ± 0.06 ^d^	15.93 ± 0.07 ^e^	2.20 ± 0.05 ^a^
Total flavan-3-ols	79.66 ± 0.36 ^a^	116.03 ± 0.36 ^f^	107.48 ± 0.38 ^c^	103.18 ± 0.41 ^b^	114.55 ± 0.44 ^d^	115.54 ± 0.46 ^e^
***Anthocyanins***						
Cy-3-Glu	nd	nd	3.77 ± 0.03 ^a^	4.40 ± 0.04 ^b^	nd	nd
Cy-3-Rut	nd	nd	nd	0.60 ± 0.02	nd	nd
Cy-3,5-diGlu	nd	10.38 ± 0.15 ^b^	nd	nd	0.10 ± 0.02 ^a^	nd
Cy-3-Xyl	nd	4.24 ± 0.04 ^b^	nd	0.17 ± 0.03 ^a^	nd	nd
Cy-3-(6″-Mal-Glu)	nd	nd	nd	0.32 ± 0.02	nd	nd
Del-3,5-diGlu	nd	7.77 ± 0.12 ^b^	nd	nd	0.03 ± 0.01 ^a^	nd
Del-3-Glu	nd	nd	nd	nd	0.01 ± 0.01	nd
Pel-3,5-diGlu	nd	10.08 ± 0.13	nd	nd	nd	nd
Total anthocyanins	nd	32.47 ± 0.24 ^d^	3.77 ± 0.03 ^b^	5.49 ± 0.11 ^c^	0.14 ± 0.04 ^a^	nd
***Phenolic acids***						
GA	6.08 ± 0.10 ^a^	7.18 ± 0.11 ^b^	7.56 ± 0.12 ^c^	7.99 ± 0.09 ^d^	7.34 ± 0.13 ^b^	8.09 ± 0.05 ^d^
PA	1.52 ± 0.04 ^a^	2.04 ± 0.05 ^c^	1.87 ± 0.03 ^b^	2.00 ± 0.03 ^c^	1.81 ± 0.05 ^b^	2.53 ± 0.10 ^d^
*p*-HBA	1.98 ± 0.06 ^a^	2.96 ± 0.07 ^c^	3.05 ± 0.02 ^d^	3.01 ± 0.05 ^d^	2.81 ± 0.04 ^b^	3.92 ± 0.04 ^e^
Total phenolic acids	9.58 ± 0.20 ^a^	12.18 ± 0.23 ^b^	12.48 ± 0.17 ^b,c^	13.00 ± 0.17 ^c^	11.96 ± 0.22 ^b^	14.54 ± 0.19 ^d^
**Total phenolics**	**89.24 ± 0.56 ^a^**	**160.68 ± 0.83 ^f^**	**123.73 ± 0.58 ^c^**	**121.67 ± 0.69 ^b^**	**126.65 ± 0.70 ^d^**	**130.08 ± 0.65 ^e^**

nd—not detected. Blueberries (BLUB), raspberries (RASB), blackberries (BLCB), pomegranates pomace (POME), beetroots (BEET), control chocolate, (CONT). Data are presented as mean ± SD of three replications. The values followed by the same lowercase letter (a–f) within each chocolate type in the same row do not differ significantly according to Tukey’s HSD test at *p* < 0.05.

**Table 4:** The content of volatile compounds in different types of chocolates.

Volatile Compounds	Functional Enrichment					
	CONT	BLUB	RASB	BLCB	POME	BEET
	**DCH**					
***Alcohols and Phenols***						
2,3-Butanediol	0.25 ± 0.04 ^a^	0.69 ± 0.05 ^e^	0.43 ± 0.03 ^c^	0.58 ± 0.06 ^d^	0.37 ± 0.08 ^b^	0.40 ± 0.02 ^c^
2-Phenylethanol	0.03 ± 0.01 ^a^	0.11 ± 0.03 ^b^	0.04 ± 0.02 ^a^	0.04 ± 0.01 ^a^	0.03 ± 0.02 ^a^	0.03 ± 0.02 ^a^
***Aldehydes and Ketones***						
2-Methylpropanal	3.49 ± 0.03 ^c^	2.84 ± 0.06 ^a^	4.09 ± 0.04 ^e^	3.68 ± 0.02 ^d^	2.99 ± 0.02 ^b^	4.66 ± 0.04 ^f^
Benzaldehyde	18.91 ± 0.13 ^d^	12.74 ± 0.14 ^a^	19.93 ± 0.10 ^e^	17.55 ± 0.15 ^c^	15.50 ± 0.13 ^b^	22.39 ± 0.19 ^f^
Butan-2-one	0.47 ± 0.06 ^b^	0.54 ± 0.02 ^c^	0.45 ± 0.03 ^b^	0.56 ± 0.04 ^c^	0.46 ± 0.06 ^b^	0.40 ± 0.02 ^a^
3-Methylbutanal	1.01 ± 0.07 ^e^	0.88 ± 0.09 ^d^	0.70 ± 0.06 ^b^	0.90 ± 0.04 ^d^	0.81 ± 0.03 ^c^	0.52 ± 0.04 ^a^
2,3-Pentanedione	0.63 ± 0.04 ^a^	1.18 ± 0.10 ^e^	0.87 ± 0.04 ^c^	1.03 ± 0.04 ^d^	0.76 ± 0.04 ^b^	0.84 ± 0.05 ^c^
Pentanal	0.08 ± 0.02 ^a^	0.67 ± 0.06 ^c^	0.29 ± 0.04 ^b^	1.13 ± 0.04 ^d^	0.11 ± 0.04 ^a^	0.09 ± 0.04 ^a^
(*Z*)-4-Heptenal	0.08 ± 0.01 ^a^	0.10 ± 0.05 ^a^	0.08 ± 0.02 ^a^	0.06 ± 0.01 ^a^	0.08 ± 0.02 ^a^	0.07 ± 0.03 ^a^
Octanal	0.02 ± 0.01 ^a^	0.10 ± 0.06 ^b^	0.02 ± 0.01 ^a^	0.02 ± 0.01 ^a^	0.01 ± 0.01 ^a^	0.01 ± 0.01 ^a^
Butanal	0.04 ± 0.01 ^b^	0.08 ± 0.03 ^c^	0.03 ± 0.01 ^a^	0.03 ± 0.01 ^a^	0.03 ± 0.01 ^a^	0.01 ± 0.01 ^a^
Nonan-2-one	0.06 ± 0.03	0.06 ± 0.01	0.07 ± 0.01	0.05 ± 0.04	0.07 ± 0.01	0.07 ± 0.02
(*Z*)-2-Nonenal	0.10 ± 0.04 ^b^	0.08 ± 0.02 ^a^	0.07 ± 0.02 ^a^	0.07 ± 0.02 ^a^	0.11 ± 0.04 ^b^	0.06 ± 0.02 ^a^
(*E*,*E*)-2,4-Nonadienal	0.02 ± 0.01 ^a^	0.09 ± 0.02 ^c^	0.06 ± 0.04 ^b^	0.05 ± 0.04 ^b^	0.03 ± 0.01 ^a^	0.07 ± 0.01 ^b^
(*Z*)-2-Decenal	0.04 ± 0.01 ^a^	0.57 ± 0.10 ^c^	0.10 ± 0.02 ^b^	0.02 ± 0.01 ^a^	0.04 ± 0.01 ^a^	0.04 ± 0.01 ^a^
Vanillin	0.06 ± 0.02	0.06 ± 0.01	0.07 ± 0.02	0.06 ± 0.01	0.06 ± 0.02	0.07 ± 0.02
***Acids***						
Pentanoic acid	0.02 ± 0.01	0.01 ± 0.01	0.02 ± 0.01	0.02 ± 0.01	0.04 ± 0.02	0.02 ± 0.01
Acetic acid	60.86 ± 0.13 ^b^	69.34 ± 0.14 ^e^	60.13 ± 0.11 ^b^	62.73 ± 0.16 ^c^	66.99 ± 0.09 ^d^	57.29 ± 0.12 ^a^
Phenylacetic acid	0.49 ± 0.02 ^d^	0.40 ± 0.03 ^b^	0.46 ± 0.01 ^c^	0.30 ± 0.02 ^a^	0.51 ± 0.03 ^d^	0.50 ± 0.04 ^d^
***Furfurals***						
2-Furfural	2.17 ± 0.04 ^d^	1.11 ± 0.05 ^a^	1.77 ± 0.07 ^b^	1.14 ± 0.03 ^a^	1.92 ± 0.05 ^c^	1.92 ± 0.04 ^c^
***Pyrazines***						
2,5-Dimethylpyrazine	10.85 ± 0.12	7.54 ± 0.11	9.80 ± 0.10	9.47 ± 0.13	8.60 ± 0.11	10.01 ± 0.12
Trimethylpyrazine	0.08 ± 0.03 ^b^	0.07 ± 0.02 ^b^	0.05 ± 0.01 ^a^	0.06 ± 0.02 ^a,b^	0.05 ± 0.02 ^a^	0.04 ± 0.01 ^a^
Tetramethylpyrazine	0.06 ± 0.01 ^a^	0.15 ± 0.05 ^c^	0.07 ± 0.02 ^a,b^	0.09 ± 0.03 ^b^	0.06 ± 0.02 ^a^	0.05 ± 0.01 ^a^
***Esters***						
Ethyl octanoate	0.03 ± 0.01 ^a^	0.05 ± 0.01 ^a^	0.07 ± 0.02 ^a,b^	0.07 ± 0.03 ^a,b^	0.07 ± 0.02 ^a,b^	0.08 ± 0.01 ^b^
Phenylethylacetate	0.13 ± 0.03 ^b^	0.31 ± 0.06 ^c^	0.16 ± 0.04 ^b^	0.07 ± 0.02 ^a^	0.13 ± 0.03 ^b^	0.16 ± 0.04 ^b^
***Lactones***						
γ-Nonalactone	nd	0.08 ± 0.02	0.08 ± 0.02	0.11 ± 0.04	0.08 ± 0.03	0.10 ± 0.05
***Sulfur Compounds***						
Dimethyl trisulfide	0.02 ± 0.01 ^a^	0.13 ± 0.03 ^c^	0.08 ± 0.01 ^b^	0.09 ± 0.02 ^b^	0.08 ± 0.03 ^b^	0.10 ± 0.04 ^b^
	**DCH98S**					
***Alcohols and Phenols***						
2,3-Butanediol	0.28 ± 0.05 ^a^	1.13 ± 0.03 ^e^	0.61 ± 0.04 ^c^	0.92 ± 0.07 ^d^	0.50 ± 0.05 ^b,c^	0.47 ± 0.03 ^b^
2-Phenylethanol	0.06 ± 0.02 ^b^	0.19 ± 0.05 ^c^	0.05 ± 0.02 ^a,b^	0.06 ± 0.02 ^b^	0.03 ± 0.01 ^a^	0.03 ± 0.01 ^a^
***Aldehydes and Ketones***						
2-Methylpropanal	3.57 ± 0.07 ^c^	2.19 ± 0.06 ^a^	4.69 ± 0.08 ^e^	3.87 ± 0.03 ^d^	2.48 ± 0.07 ^b^	5.24 ± 0.08 ^f^
Benzaldehyde	17.34 ± 0.15 ^d^	6.58 ± 0.09 ^a^	20.95 ± 0.17 ^e^	16.18 ± 0.09 ^c^	12.09 ± 0.11 ^b^	24.13 ± 0.18 ^f^
Butan-2-one	0.35 ± 0.04 ^a^	0.60 ± 0.07 ^c^	0.43 ± 0.05 ^b^	0.65 ± 0.06 ^c^	0.46 ± 0.03 ^b^	0.36 ± 0.05 ^a^
3-Methylbutanal	0.51 ± 0.03 ^c^	0.75 ± 0.05 ^e^	0.40 ± 0.06 ^b^	0.80 ± 0.05 ^e^	0.61 ± 0.04 ^d^	0.28 ± 0.03 ^a^
2,3-Pentanedione	0.93 ± 0.07 ^b^	1.72 ± 0.08 ^e^	1.10 ± 0.05 ^c^	1.42 ± 0.09 ^d^	0.88 ± 0.08 ^a^	0.93 ± 0.07 ^b^
Pentanal	0.13 ± 0.06 ^b^	1.26 ± 0.07 ^d^	0.50 ± 0.03 ^c^	2.18 ± 0.04 ^e^	0.13 ± 0.03 ^b^	0.09 ± 0.03 ^a^
(*Z*)-4-Heptenal	0.06 ± 0.02 ^b^	0.11 ± 0.05 ^c^	0.07 ± 0.02 ^b^	0.04 ± 0.01 ^a^	0.07 ± 0.02 ^b^	0.07 ± 0.02 ^b^
Octanal	0.02 ± 0.01 ^a^	0.18 ± 0.03 ^b^	0.03 ± 0.01 ^a^	0.03 ± 0.01 ^a^	0.01 ± 0.01 ^a^	0.01 ± 0.01 ^a^
Butanal	0.02 ± 0.01 ^a^	0.13 ± 0.04 ^b^	0.02 ± 0.01 ^a^	0.03 ± 0.01 ^a^	0.03 ± 0.01 ^a^	0.01 ± 0.01 ^a^
Nonan-2-one	0.02 ± 0.01 ^a^	0.07 ± 0.02 ^b^	0.07 ± 0.01 ^b^	0.04 ± 0.02 ^a^	0.08 ± 0.02 ^b^	0.08 ± 0.03 ^b^
(*Z*)-2-Nonenal	0.02 ± 0.01 ^a^	0.06 ± 0.02 ^b^	0.04 ± 0.02 ^a,b^	0.02 ± 0.01 ^a^	0.12 ± 0.04 ^c^	0.03 ± 0.01 ^a^
(*E*,*E*)-2,4-Nonadienal	0.12 ± 0.05 ^c^	0.16 ± 0.06 ^d^	0.11 ± 0.03 ^b,c^	0.08 ± 0.02 ^b^	0.03 ± 0.01 ^a^	0.10 ± 0.03 ^b,c^
(*Z*)-2-Decenal	0.03 ± 0.01 ^a^	1.10 ± 0.04 ^d^	0.17 ± 0.05 ^c^	0.02 ± 0.01 ^a^	0.04 ± 0.01 ^a,b^	0.05 ± 0.02 ^b^
Vanillin	0.06 ± 0.02	0.06 ± 0.02	0.07 ± 0.02	0.06 ± 0.02	0.06 ± 0.01	0.07 ± 0.03
***Acids***						
Pentanoic acid	0.02 ± 0.01 ^a^	0.01 ± 0.01 ^a^	0.02 ± 0.01 ^a^	0.02 ± 0.01 ^a^	0.06 ± 0.02 ^b^	0.03 ± 0.01 ^a^
Acetic acid	67.02 ± 0.12 ^d^	77.90 ± 0.15 ^f^	59.48 ± 0.11 ^b^	64.68 ± 0.10 ^c^	73.20 ± 0.13 ^e^	55.54 ± 0.16 ^a^
Phenylacetic acid	0.02 ± 0.01 ^a^	0.32 ± 0.05 ^c^	0.43 ± 0.04 ^d^	0.10 ± 0.03 ^b^	0.53 ± 0.07 ^e^	0.50 ± 0.06 ^e^
***Furfurals***						
2-Furfural	1.63 ± 0.04 ^d^	0.05 ± 0.01 ^a^	1.36 ± 0.09 ^c^	0.11 ± 0.03 ^b^	1.68 ± 0.07 ^d^	1.79 ± 0.05 ^e^
*Pyrazines*						
2,5-Dimethylpyrazine	7.60 ± 0.10 ^c^	4.14 ± 0.09 ^a^	8.67 ± 0.07 ^e^	8.01 ± 0.08 ^d^	6.28 ± 0.11 ^b^	9.54 ± 0.14 ^f^
Trimethylpyrazine	0.02 ± 0.01 ^a^	0.06 ± 0.02 ^b^	0.03 ± 0.01 ^a^	0.04 ± 0.02 ^a^	0.03 ± 0.01 ^a^	0.03 ± 0.01 ^a^
Tetramethylpyrazine	0.06 ± 0.02 ^a^	0.25 ± 0.06 ^d^	0.09 ± 0.03 ^b^	0.13 ± 0.04 ^c^	0.06 ± 0.02 ^a^	0.05 ± 0.02 ^a^
***Esters***						
Ethyl octanoate	0.03 ± 0.01 ^a^	0.08 ± 0.02 ^b^	0.10 ± 0.03 ^c^	0.12 ± 0.04 ^c^	0.11 ± 0.03 ^c^	0.11 ± 0.02 ^c^
Phenylethylacetate	0.02 ± 0.01 ^a^	0.49 ± 0.07 ^d^	0.19 ± 0.05 ^c^	0.02 ± 0.01 ^a^	0.13 ± 0.03 ^b^	0.17 ± 0.05 ^c^
***Lactones***						
γ-Nonalactone	nd	0.16 ± 0.03 ^a^	0.16 ± 0.05 ^a^	0.21 ± 0.07 ^b^	0.16 ± 0.05 ^a^	0.15 ± 0.03 ^a^
***Sulfur Compounds***						
Dimethyl trisulfide	0.08 ± 0.03 ^a^	0.25 ± 0.06 ^c^	0.15 ± 0.02 ^b^	0.16 ± 0.03 ^b^	0.15 ± 0.04 ^b^	0.13 ± 0.02 ^b^
	**DCH98ESt**					
***Alcohols and Phenols***						
2,3-Butanediol	0.29 ± 0.05 ^a^	0.71 ± 0.06 ^d^	0.53 ± 0.04 ^b^	0.60 ± 0.03 ^c^	0.51 ± 0.03 ^b^	0.50 ± 0.04 ^b^
2-Phenylethanol	0.06 ± 0.02 ^b^	0.12 ± 0.03 ^c^	0.04 ± 0.02 ^a^	0.06 ± 0.02 ^b^	0.04 ± 0.01 ^a^	0.03 ± 0.01 ^a^
***Aldehydes And Ketones***						
2-Methylpropanal	2.78 ± 0.04 ^b^	2.48 ± 0.04 ^a^	4.14 ± 0.06 ^d^	3.32 ± 0.04 ^c^	3.31 ± 0.05 ^c^	4.69 ± 0.05 ^e^
Benzaldehyde	17.67 ± 0.11 ^d^	12.12 ± 0.10 ^a^	19.05 ± 0.09 ^e^	16.93 ± 0.11 ^c^	15.57 ± 0.12 ^b^	21.59 ± 0.12 ^f^
Butan-2-one	0.31 ± 0.02 ^a^	0.46 ± 0.03 ^c^	0.42 ± 0.04 ^b^	0.48 ± 0.04 ^c^	0.44 ± 0.05 ^b,c^	0.39 ± 0.04 ^b^
3-Methylbutanal	0.43 ± 0.03 ^b^	0.59 ± 0.03 ^d^	0.43 ± 0.03 ^b^	0.61 ± 0.04 ^d^	0.52 ± 0.05 ^c^	0.35 ± 0.03 ^a^
2,3-Pentanedione	1.48 ± 0.03 ^c^	1.60 ± 0.04 ^d^	0.98 ± 0.03 ^b^	1.45 ± 0.04 ^c^	0.93 ± 0.04 ^a^	0.95 ± 0.04 ^a,b^
Pentanal	1.59 ± 0.05 ^d^	1.42 ± 0.04 ^c^	0.24 ± 0.03 ^b^	1.89 ± 0.05 ^e^	0.18 ± 0.04 ^a^	0.17 ± 0.03 ^a^
(*Z*)-4-Heptenal	0.04 ± 0.01 ^a^	0.08 ± 0.04 ^b^	0.07 ± 0.04 ^b^	0.04 ± 0.01 ^a^	0.07 ± 0.04 ^b^	0.07 ± 0.02 ^b^
Octanal	0.02 ± 0.01 ^a^	0.10 ± 0.02 ^b^	0.01 ± 0.01 ^a^	0.02 ± 0.01 ^a^	0.01 ± 0.01 ^a^	0.01 ± 0.01 ^a^
Butanal	0.02 ± 0.01 ^a^	0.08 ± 0.02 ^b^	0.02 ± 0.01 ^a^	0.03 ± 0.01 ^a^	0.02 ± 0.01 ^a^	0.01 ± 0.01 ^a^
Nonan-2-one	0.02 ± 0.01 ^a^	0.04 ± 0.01 ^a^	0.08 ± 0.01 ^b^	0.03 ± 0.01 ^a^	0.08 ± 0.01 ^b^	0.08 ± 0.02 ^b^
(*Z*)-2-Nonenal	0.02 ± 0.01 ^a^	0.04 ± 0.01 ^a,b^	0.06 ± 0.01 ^a^	0.03 ± 0.01^a^	0.09 ± 0.02 ^c^	0.05 ± 0.01 ^a^
(*E*,*E*)-2,4-Nonadienal	0.03 ± 0.01 ^a^	0.10 ± 0.03 ^b^	0.08 ± 0.02 ^b^	0.05 ± 0.02 ^a^	0.05 ± 0.01 ^a^	0.09 ± 0.01 ^b^
(*Z*)-2-Decenal	0.04 ± 0.01 ^a^	0.57 ± 0.05 ^c^	0.09 ± 0.02 ^b^	0.02 ± 0.01 ^a^	0.07 ± 0.02 ^b^	0.07 ± 0.02 ^b^
Vanillin	0.06 ± 0.01	0.06 ± 0.01	0.07 ± 0.02	0.06 ± 0.02	0.06 ± 0.01	0.07 ± 0.01
***Acids***						
Pentanoic acid	0.02 ± 0.01 ^a^	0.01 ± 0.01 ^a^	0.04 ± 0.01 ^a,b^	0.01 ± 0.01 ^a^	0.05 ± 0.01 ^b^	0.03 ± 0.01 ^a^
Acetic acid	67.46 ± 0.14 ^d^	72.69 ± 0.15 ^e^	62.74 ± 0.17 ^b^	66.08 ± 0.15 ^c^	67.97 ± 0.17 ^d^	59.14 ± 0.16 ^a^
Phenylacetic acid	0.03 ± 0.01 ^a^	0.17 ± 0.03 ^b^	0.49 ± 0.04 ^c^	0.07 ± 0.01 ^b^	0.51 ± 0.06 ^c^	0.49 ± 0.05 ^c^
***Furfurals***						
2-Furfural	0.06 ± 0.01 ^a^	0.05 ± 0.01 ^a^	1.61 ± 0.06 ^b^	0.08 ± 0.02 ^a^	1.64 ± 0.05 ^b^	1.70 ± 0.08 ^c^
***Pyrazines***						
2,5-Dimethylpyrazine	7.38 ± 0.13 ^b^	5.69 ± 0.12 ^a^	8.16 ± 0.14 ^d^	7.62 ± 0.12 ^c^	7.22 ± 0.12 ^b^	8.85 ± 0.13 ^e^
Trimethylpyrazine	0.02 ± 0.01	0.04 ± 0.01	0.03 ± 0.01	0.03 ± 0.01	0.03 ± 0.01	0.03 ± 0.01
Tetramethylpyrazine	0.06 ± 0.01 ^a^	0.15 ± 0.03 ^c^	0.07 ± 0.02 ^a^	0.09 ± 0.01 ^a,b^	0.06 ± 0.01 ^a^	0.06 ± 0.01 ^a^
***Esters***						
Ethyl octanoate	0.02 ± 0.01 ^a^	0.05 ± 0.01 ^b^	0.11 ± 0.03 ^c^	0.07 ± 0.01 ^b^	0.11 ± 0.02 ^c^	0.11 ± 0.01 ^c^
Phenylethylacetate	0.03 ± 0.01 ^a^	0.26 ± 0.07 ^c^	0.16 ± 0.03 ^b^	0.02 ± 0.01 ^a^	0.15 ± 0.03 ^b^	0.17 ± 0.04 ^b^
***Lactones***						
γ-Nonalactone	nd	0.15 ± 0.02	0.16 ± 0.03	0.18 ± 0.04	0.16 ± 0.03	0.15 ± 0.03
***Sulfur Compounds***						
Dimethyl trisulfide	0.07 ± 0.02 ^a^	0.16 ± 0.03 ^b^	0.14 ± 0.03 ^b^	0.12 ± 0.02 ^b^	0.15 ± 0.03 ^b^	0.14 ± 0.02 ^b^
	**MCH**					
***Alcohols and Phenols***						
2,3-Butanediol	0.29 ± 0.03 ^a^	0.79 ± 0.06 ^e^	0.53 ± 0.06 ^d^	0.58 ± 0.08 ^d^	0.36 ± 0.04 ^b^	0.41 ± 0.03 ^c^
2-Phenylethanol	0.03 ± 0.01 ^a^	0.10 ± 0.02 ^b^	0.05 ± 0.01 ^a^	0.04 ± 0.01 ^a^	0.03 ± 0.01 ^a^	0.03 ± 0.01 ^a^
***Aldehydes and Ketones***						
2-Methylpropanal	3.42 ± 0.07 ^b^	2.83 ± 0.05 ^a^	4.37 ± 0.07 ^c^	3.41 ± 0.06 ^b^	3.10 ± 0.04 ^b^	4.63 ± 0.07 ^d^
Benzaldehyde	17.96 ± 0.10 ^c^	11.88 ± 0.08 ^a^	20.20 ± 0.12 ^d^	16.08 ± 0.11 ^b^	16.00 ± 0.09 ^b^	22.07 ± 0.10 ^e^
Butan-2-one	0.45 ± 0.04 ^b^	0.55 ± 0.05 ^c^	0.43 ± 0.05 ^b^	0.53 ± 0.06 ^c^	0.45 ± 0.04 ^b^	0.39 ± 0.03 ^a^
3-Methylbutanal	0.69 ± 0.05 ^c,d^	0.72 ± 0.06 ^d^	0.47 ± 0.05 ^b^	0.71 ± 0.07 ^d^	0.66 ± 0.07 ^c^	0.41 ± 0.05 ^a^
2,3-Pentanedione	1.22 ± 0.06 ^c^	1.40 ± 0.07 ^d^	1.13 ± 0.06 ^b^	1.24 ± 0.07 ^c^	1.11 ± 0.06 ^b^	1.04 ± 0.05 ^a^
Pentanal	0.30 ± 0.02 ^c^	1.03 ± 0.05 ^e^	0.45 ± 0.03 ^d^	1.02 ± 0.06 ^e^	0.24 ± 0.03 ^b^	0.16 ± 0.03 ^a^
(*Z*)-4-Heptenal	0.11 ± 0.03 ^b^	0.09 ± 0.03 ^a^	0.08 ± 0.02 ^a^	0.07 ± 0.04 ^a^	0.09 ± 0.03 ^a^	0.08 ± 0.02 ^a^
Octanal	0.02 ± 0.01 ^a^	0.08 ± 0.04 ^b^	0.02 ± 0.01 ^a^	0.02 ± 0.01 ^a^	0.01 ± 0.01 ^a^	0.01 ± 0.01 ^a^
Butanal	0.03 ± 0.01 ^a^	0.07 ± 0.04 ^b^	0.02 ± 0.01 ^a^	0.03 ± 0.01 ^a^	0.03 ± 0.01 ^a^	0.01 ± 0.01 ^a^
Nonan-2-one	0.06 ± 0.03	0.06 ± 0.03	0.07 ± 0.03	0.06 ± 0.04	0.07 ± 0.04	0.07 ± 0.03
(*Z*)-2-Nonenal	0.11 ± 0.04 ^c^	0.08 ± 0.03 ^a,b^	0.06 ± 0.03 ^a^	0.08 ± 0.04 ^a,b^	0.11 ± 0.04 ^c^	0.06 ± 0.03 ^a^
(*E*,*E*)-2,4-Nonadienal	0.02 ± 0.01 ^a^	0.09 ± 0.04 ^b^	0.08 ± 0.03 ^b^	0.04 ± 0.01 ^a^	0.02 ± 0.01 ^a^	0.07 ± 0.03 ^b^
(*Z*)-2-Decenal	0.15 ± 0.05 ^b^	0.48 ± 0.07 ^c^	0.17 ± 0.04 ^b^	0.07 ± 0.02 ^a^	0.11 ± 0.06 ^a^	0.08 ± 0.03 ^a^
Vanillin	0.06 ± 0.02	0.06 ± 0.02	0.07 ± 0.04	0.06 ± 0.04	0.06 ± 0.03	0.07 ± 0.04
***Acids***						
Pentanoic acid	0.05 ± 0.01	0.03 ± 0.01	0.03 ± 0.01	0.04 ± 0.02	0.05 ± 0.03	0.04 ± 0.02
Acetic acid	64.84 ± 0.17 ^b^	71.71 ± 0.19 ^e^	60.82 ± 0.15 ^a^	66.45 ± 0.16 ^c^	67.64 ± 0.15 ^d^	58.64 ± 0.12 ^a^
Phenylacetic acid	0.15 ± 0.04 ^a^	0.29 ± 0.04 ^c^	0.36 ± 0.05 ^b^	0.21 ± 0.04 ^b^	0.28 ± 0.04 ^c^	0.38 ± 0.05 ^b^
***Furfurals***						
2-Furfural	1.93 ± 0.08 ^e^	0.76 ± 0.07 ^a^	1.51 ± 0.08 ^c^	1.15 ± 0.07 ^b^	1.85 ± 0.09 ^d^	1.84 ± 0.04 ^d^
***Pyrazines***						
2,5-Dimethylpyrazine	7.44 ± 0.12 ^c^	6.00 ± 0.11 ^a^	8.37 ± 0.11 ^d^	7.44 ± 0.10 ^c^	7.06 ± 0.09 ^b^	8.83 ± 0.12 ^e^
Trimethylpyrazine	0.02 ± 0.01	0.03 ± 0.01	0.03 ± 0.01	0.03 ± 0.01	0.02 ± 0.01	0.02 ± 0.01
Tetramethylpyrazine	0.06 ± 0.01 ^a^	0.16 ± 0.03 ^b^	0.08 ± 0.01 ^a^	0.08 ± 0.02 ^a^	0.06 ± 0.02 ^a^	0.05 ± 0.01 ^a^
***Esters***						
Ethyl octanoate	0.03 ± 0.01	0.08 ± 0.01 ^b^	0.08 ± 0.02 ^b^	0.08 ± 0.02 ^b^	0.05 ± 0.01 ^a^	0.08 ± 0.01 ^b^
Phenylethylacetate	0.49 ± 0.05 ^d^	0.32 ± 0.04 ^b,c^	0.26 ± 0.04 ^b^	0.22 ± 0.04 ^a^	0.37 ± 0.05 ^c^	0.28 ± 0.04 ^b^
***Lactones***						
γ-Nonalactone	nd	0.14 ± 0.03 ^b^	0.12 ± 0.02 ^b^	0.12 ± 0.01 ^b^	0.05 ± 0.01 ^a^	0.11 ± 0.02 ^b^
***Sulfur Compounds***						
Dimethyl trisulfide	0.08 ± 0.01 ^a^	0.18 ± 0.02 ^c^	0.13 ± 0.03 ^b^	0.13 ± 0.02 ^b^	0.10 ± 0.01 ^a,b^	0.12 ± 0.02 ^b^

nd—not detected. Blueberries (BLUB), raspberries (RASB), blackberries (BLCB), pomegranates pomace (POME), beetroots (BEET), control (CONT). Data are expressed as the relative peak area (in percentage) of each compound and presented as mean ± SD of three replications. The values followed by the same lowercase letter (a–f) within each chocolate type in the same row do not differ significantly according to Tukey’s HSD test at *p* < 0.05.

**Table 5:** Recipes of examined chocolates.

Raw Material	Content (%)							
	Control DCH	DCH	Control DCH98S	DCH98S	Control DCH98ESt	DCH98ESt	Control MCH	MCH
Cocoa liquor	40.00	40.00	92.00	92.00	92.00	92.00	20.00	20.00
Milk powder	-	-	-	-	-	-	20.00	20.00
Cocoa butter	13.40	13.40	0.80	0.80	0.80	0.80	19.80	19.80
Alkalized cocoa powder	-	-	5.00	5.00	5.00	5.00	-	-
Lecithin	0.50	0.50	0.50	0.50	0.50	0.50	0.50	0.50
PGPR	0.50	0.50	0.50	0.50	0.50	0.50	0.50	0.50
Ethyl vanillin	0.01	0.01	0.01	0.01	0.01	0.01	0.01	0.01
Sugar (sucrose)	45.59	44.59	1.19	0.19	-	-	39.19	38.19
Erythritol+stevia	-	-	-	-	1.19	0.19	-	-
Lyophilizate of fruits or vegetables	-	1.00	-	1.00	-	1.00	-	1.00

DCH—dark chocolate with 53% cocoa content sweetened with sucrose, DCH98S—dark chocolate with 98% cocoa content sweetened with sucrose, DCH98ESt—dark chocolate with 98% cocoa content sweetened with erythritol with stevia, MCH—milk chocolate with 40% cocoa content sweetened with sucrose.

## Data Availability

All the data are included in the present study.

## Supplementary Materials

The following are available online, Table S1: Two-way ANOVA analysis of physicochemical characteristic and organoleptic assessment of different types of chocolates enriched with various freeze-dried phenolic-rich plant powders; Table S2: Two-way ANOVA analysis of the content of individual phenolic compounds and antioxidant properties of different types of chocolates enriched with various freeze-dried phenolic-rich plant powders; Table S3: Two-way ANOVA analysis of the content of volatile compounds in different types of chocolates enriched with various freeze-dried phenolic-rich plant powders.

## Abbreviations

BEET	beetroots
BLCB	blackberries
BLUB	blueberries
Cat	catechin
CONT	control chocolates without the addition of freeze-dried phenolic-rich plant powders
Cy-3-(6″-Mal-Glu)	cyanidin-3-(6″-malonyl)-glucoside
Cy-3,5-diGlu	cyanidin-3,5-*O*-diglucoside
Cy-3-Glu	cyanidin-3-*O*-glucoside
Cy-3-Rut	cyanidin-3-*O*-rutinoside
Cy-3-Xyl	cyanidin-3-*O*-xyloside
DCH	dark chocolate with 53% cocoa and the total fat content of 35% (*w*/*w*) sweetened with sucrose
DCH98Est	dark chocolate with 98% cocoa and the total fat content of 51% (*w*/*w*) sweetened with erythritol with stevia
DCH98S	dark chocolate with 98% cocoa and the total fat content of 51% (*w*/*w*) sweetened with sucrose
Del-3,5-diGlu	delphinidin-3,5-*O*-diglucoside
Del-3-Glu	delphinidin-3-*O*-glucoside
Ecat	epicatechin
GA	gallic acid
MCH	milk chocolate with the total fat content of 36% (*w*/*w*) sweetened with sucrose
PA	protocatechuic acid
PC B2	procyanidin B2
PC C1	procyanidin C1
PCA	principal component analysis
Pel-3,5-diGlu	pelargonidin-3,5-*O*-diglucoside
*p*-HBA	*p*-hydroxybenzoic acid
POME	pomegranates pomace
RASP	raspberries
VCs	volatile compounds
