# Supplementary material for: Influence of Freeze-Dried Phenolic-Rich Plant Powders on the Bioactive Compounds Profile, Antioxidant Activity and Aroma of Different Types of Chocolates
Source: Molecules. 2021 Nov 22;26(22):7058. doi: 10.3390/molecules26227058 (PMC8622861; doi:10.3390/molecules26227058)
Supplement: Supplementary file 1 [file molecules-26-07058-s001.zip › molecules-1437282-supplementary.pdf]

molecules

MDPI

Supplementary Materials

Influence of Freeze-Dried Phenolic-Rich Plant Powders on the Bioactive Compounds Profile, Antioxidant Activity and Aroma of Different Types of Chocolates

Dorota Żyżelewicz [1,*], Joanna Oracz [1], Martyna Bilicka [1], Kamila Kulbat-Warycha [1] and Elżbieta Klewicka [2]

1 Institute of Food Technology and Analysis, Faculty of Biotechnology and Food Sciences, Lodz University of Technology, 2/22 Stefanowskiego Street, 90-537 Łódź, Poland; joanna.oracz@p.lodz.pl (J.O.); martyna.bilicka@gmail.com (M.B.); kamila.kulbat-warycha@p.lodz.pl (K.K.-W.)
2 Institute of Fermentation Technology and Microbiology, Faculty of Biotechnology and Food Sciences, Lodz University of Technology, 171/173 Wólczańska Street, 90-530 Łódź, Poland; elzbieta.klewicka@p.lodz.pl
* Correspondence: dorota.zyzelewicz@p.lodz.pl; Tel.: +48-42-631-34-61

TABLE OF CONTENTS

Tables

Table S1 .... S2
Table S2 .... S3
Table S3 .... S4

Table S1. Two-way ANOVA analysis of physicochemical characteristic and organoleptic assessment of different types of chocolates enriched with various freeze-dried phenolic-rich plant powders.

ANOVA Factor	Water Content (%)	Water Activity	CIE L*a*b* Color Parameters			Organoleptic Assessment (Point)
			L*	a*	b*	
**Chocolate type (ChT)**						
p value	***	***	***	***	***	***
**Phenolic-rich plant enrichment (PE)**						
p value	***	***	*	***	***	***
**ChT × PE interaction**						
p value	***	***	**	**	***	**

NS = not significant ($p > 0.05$), * $p < 0.05$, ** $p < 0.01$, *** $p < 0.001$.

Table S2. Two-way ANOVA analysis of the content of individual phenolic compounds and antioxidant properties of different types of chocolates enriched with various freeze-dried phenolic-rich plant powders.

Parameters	*p* value		
	**Chocolate Type (ChT)**	**Phenolic-Rich Plant Enrichment (PE)**	**ChT × PE Interaction**
**Phenolic content**			
Cat	***	***	***
Ecat	***	***	***
PC B2	***	***	***
PC C1	***	***	***
Cy-3-Glu	NS	***	NS
Cy-3-Rut	NS	***	NS
Cy-3,5-diGlu	NS	***	NS
Cy-3-Xyl	NS	***	NS
Cy-3-(6''-Mal-Glu)	NS	***	NS
Del-3,5-diGlu	NS	***	NS
Del-3-Glu	NS	***	NS
Pel-3,5-diGlu	***	***	***
GA	***	***	***
PA	***	***	***
p-HBA	***	***	***
Total flavan-3-ols	***	***	***
Total anthocyanins	NS	***	NS
Total phenolic acids	***	***	***
Total phenolics	***	***	***
Antioxidant activity			
DPPH EC_{50}	***	***	***
FRAP	***	***	***

NS = not significant ($p > 0.05$), * $p < 0.05$, ** $p < 0.01$, *** $p < 0.001$.

Table S3. Two-way ANOVA analysis of the content of volatile compounds in different types of chocolates enriched with various freeze-dried phenolic-rich plant powders.

Parameters	*p* value		
	**Chocolate Type (ChT)**	**Phenolic-Rich Plant Enrichment (PE)**	**ChT × PE Interaction**
Alcohols and phenols			
2,3-Butanediol	***	***	***
2-Phenylethanol	***	***	***
Aldehydes and ketones			
2-Methylpropanal	***	***	***
Benzaldehyde	***	***	***
Butan-2-one	***	***	***
3-Methylbutanal	***	***	***
2,3-Pentanedione	***	***	***
Pentanal	***	***	***
(*Z*)-4-Heptenal	***	***	***
Octanal	***	***	***
Butanal	***	***	***
Nonan-2-one	***	***	***
(*Z*)-2-Nonenal	***	***	***
(*E*,*E*)-2,4-Nonadienal	***	***	***
(*Z*)-2-Decenal	***	***	***
Vanillin	NS	***	NS
Acids			
Pentanoic acid	***	***	***
Acetic acid	***	***	***
Phenylacetic acid	***	***	***
Furfurals			
2-Furfural	***	***	***
Pyrazines			
2,5-Dimethylpyrazine	***	***	***
Trimethylpyrazine	***	***	***
Tetramethylpyrazine	***	***	***
Esters			
Ethyl octanoate	***	***	***
Phenylethylacetate	***	***	***
Lactones			
γ-Nonalactone	***	***	***
Sulfur compounds			
Dimethyl trisulfide	***	***	***

NS = not significant ($p > 0.05$), * $p < 0.05$, ** $p < 0.01$, *** $p < 0.001$.
